# Fe_3_O_4_@SiO_2_-LY-C-D-Pd as a new, effective, and magnetically recoverable catalyst for the synthesis of 1H-tetrazoles and asymmetric biphenyls

**DOI:** 10.1038/s41598-025-95922-x

**Published:** 2025-04-15

**Authors:** Shelesh Krishna Saraswat, Ahmed M. Naglah, Jayanti Makasana, Hamidah Abu Bakar, Suhas Ballal, Munthar Kadhim Abosaoda, V. Kavitha, Lakshay Bareja, Pushpa Negi Bhakuni, Ojas Prakashbhai Doshi

**Affiliations:** 1 Department of Electronics and Communication Engineering, GLA University, Mathura, 281406 India; 2 Department of Pharmaceutical Chemistry, College of Pharmacy, King Saud University, P.O. BOX 2457, 11451 Riyadh, Saudi Arabia; 3 Marwadi University Research Center, Department of Chemistry, Faculty of Science, Marwadi University, Rajkot, Gujarat 360003 India; 4 Management and Science University, Shah Alam, Malaysia; 5 Department of Chemistry and Biochemistry, School of Sciences, JAIN (Deemed to be University), Bangalore, Karnataka India; 6 College of Pharmacy, The Islamic University, Najaf, Iraq; 7 College of Pharmacy, The Islamic University of Al Diwaniyah, Al Diwaniyah, Iraq; 8 Department of Chemistry, Sathyabama Institute of Science and Technology, Chennai, Tamil Nadu India; 9 Centre for Research Impact and Outcome, Chitkara University Institute of Engineering and Technology, Chitkara University, Rajpura, Punjab 140401 India; 10 Department of Allied Science, Graphic Era Hill University, Bhimtal, Uttarakhand 248002 India; 11 Graphic Era Deemed to be University, Dehradun, Uttarakhand India; 12 Arnold and Marie Schwartz College of Pharmacy and Health Sciences, Long Island University, Brooklyn, NY USA

**Keywords:** Complex, Fe_3_O_4_, Coupling, Catalyst, Catalysis, Catalyst synthesis, Catalytic mechanisms, Organocatalysis

## Abstract

This research project explored the synthesis and characterization of a newly developed C-D-Pd complex immobilized on Fe_3_O_4_@SiO_2_-LY, designed as a reusable magnetic catalyst. The heterogeneous nanocatalyst was thoroughly characterized using EDS, FTIR, XRD, XPS, TGA, SEM, VSM, and ICP techniques. The Fe_3_O_4_@SiO_2_-LY-C-D-Pd catalyst demonstrates exceptional performance in catalyzing C–C coupling reactions and ^1^H-tetrazole derivatives, achieving high product yields. This catalyst offers several advantages, including eco-friendly reaction conditions, minimal catalyst usage, a simple experimental setup, the elimination of harmful organic solvents, reduced reaction times, and the ability to accommodate diverse substrates. Additionally, the nanocatalyst is easily separable from the reaction mixture and can be reused multiple times without losing stability or catalytic efficiency.

## Introduction

In recent years, synthetic organic chemistry has advanced significantly, placing greater emphasis on the efficient isolation, purification, and reusability of reagents and catalysts, particularly from an industrial and eco-friendly perspective^1–3^. Even trace contamination of a transition metal catalyst in the final product can lead to toxicity, and its removal often involves conventional methods that not only raise production costs but also complicate regulatory compliance both of which are undesirable in industrial processes^4–7^. To address this challenge, magnetic nanoparticles (MNPs) have gained prominence as pseudo-heterogeneous supports for catalytic applications. These materials eliminate the need for traditional separation techniques such as centrifugation, filtration, or extraction, as they can instead be retrieved using an external magnet^8,9^. Their small size and high surface-to-volume ratio make them ideal for surface loading while offering catalytic performance on par with their homogeneous counterparts^10,11^. The development of magnetic nanoparticles has recently emerged as a promising approach in the synthesis of organic compounds^12,13^. This advancement effectively addresses the challenge of solid loss that commonly occurs during the recovery of heterogeneous catalysts via filtration^14^. However, designing efficient nanomaterials as catalyst supports remains a critical hurdle in modern organic synthesis^15^. Over the last decade, magnetic nanoparticles have become widely utilized as catalyst supports because of their straightforward preparation, easy recovery using magnetic fields, and high surface area, all of which help reduce operating costs^16,17^. Among these, Fe_3_O_4_ has gained prominence as a heterogeneous catalyst due to its simple synthesis and magnet-enabled surface modification^18^. Additionally, when coated with silica, Fe_3_O_4_ nanoparticles offer enhanced thermal stability, increased surface area for the immobilization of organic ligands, and facilitate easy recovery and reuse across multiple reaction cycles^19–21^. Among the various reported methods for C–C coupling in organic reactions, transition-metal-catalyzed processes particularly those involving palladium (Pd) have emerged in recent years as atom- and step-economical tools for constructing diverse organic molecules^22,23^. Among these, the Suzuki cross-coupling reaction stands out as a widely recognized, highly significant, and versatile methodology for achieving C–C bond formation in organic synthesis. Notably, Pd-complexes incorporating electron-rich and sterically hindered Cyanuric chloride-Dipyridilamine (C-D) ligands have proven to be highly effective catalysts for sterically challenging Suzuki couplings. These ligands are thought to stabilize a putative monoligated Pd-complex, enhancing their catalytic efficiency^24–28^. Tetrazoles are significant heterocyclic compounds with a wide range of applications across various fields, including organic synthesis, material science, coordination chemistry, and organometallic chemistry^29,30^. They serve as effective stabilizers for metallopeptide structures, and stable surrogates for carboxylic acids, and play a crucial role in medicinal chemistry. Prominent examples of their use in pharmaceuticals include the drugs valsartan and losartan. More recently, tetrazole derivatives have been employed to facilitate the binding of aryl thiotetrazolylacetanilides with HIV-1 reverse transcriptase. Tetrazoles and their derivatives demonstrate versatile biological activities, being reported as antiviral, antibacterial, anti-inflammatory, and herbicidal agents^31^. They also show promise as potential anti-HIV drug candidates, as well as exhibiting anti-proliferative, analgesic, and antitumor properties^32^. In drug design, tetrazoles are often utilized as isosteric replacements for carboxylic acids. Additionally, they have found applications in information storage systems. The conventional method for synthesizing 5-substituted 1H-tetrazoles involves the [3 + 2] cycloaddition of azides to nitriles^33^. While numerous procedures are based on this approach, many suffer from notable drawbacks^34^. These include the use of organic solvents, harsh reaction conditions, sensitivity to water, extended reaction times, and challenges in catalyst separation and recovery^35^. Consequently, the development of more efficient and sustainable methods for synthesizing these heterocycles has attracted significant research interest.

In this report, we present the synthesis of an effective heterogeneous catalyst, C-D-Pd (Cyanuric chloride-Dipyridilamine-Pd), coated on Fe_3_O_4_ MNPs, and its application in achieving high yields in the synthesis of 1H-tetrazoles and asymmetric biphenyls under mild conditions. This development harnesses the potential benefits of using novel and environmentally friendly materials in heterogeneous catalysis.

## Experimental

### Materials and characterization

The materials and solvents used in this study were obtained from Sigma-Aldrich, Fluka, or Merck and utilized directly without further purification.

### Preparation of Fe_3_O_4_@SiO_2_-LY-C-D-Pd

**Fig. 1:**
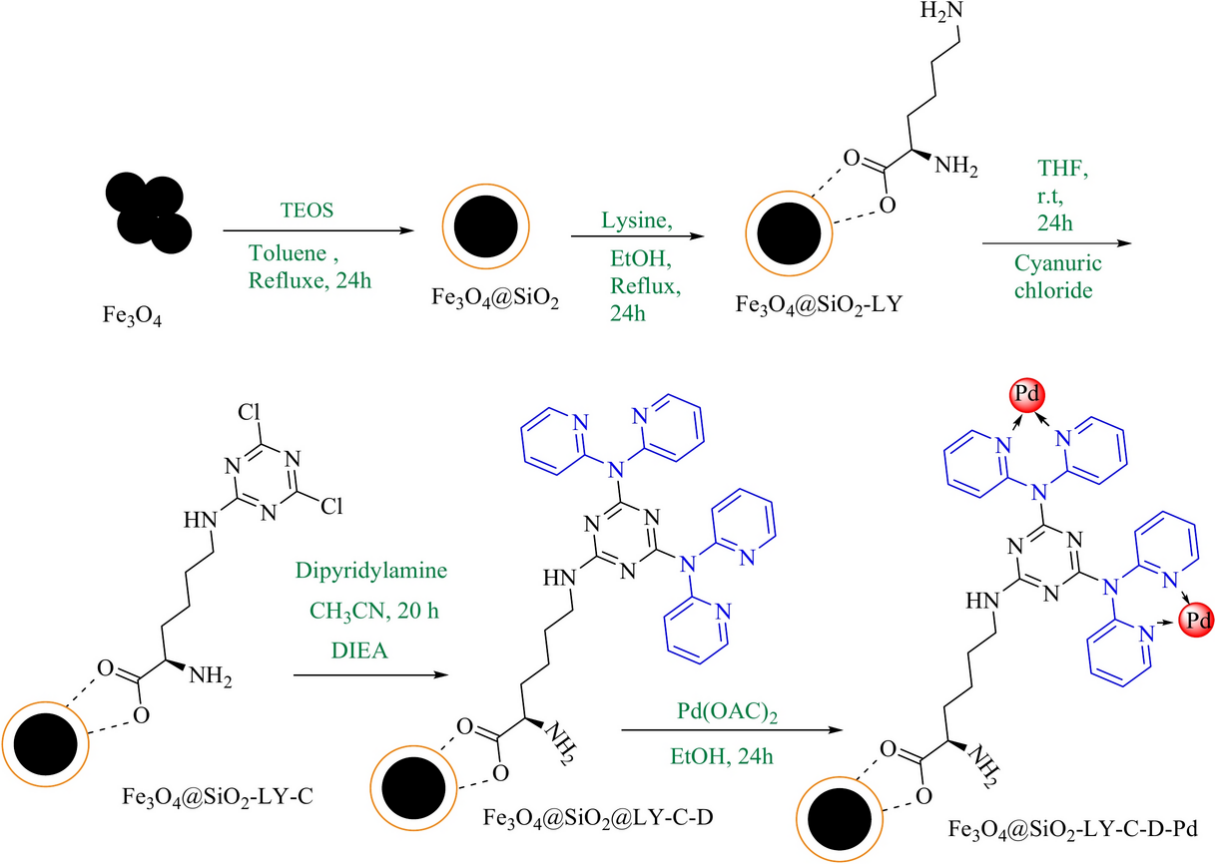
Synthetic route for Fe_3_O_4_@SiO_2_-LY-C-D-Pd.

The synthesis process began with the preparation of Fe_3_O_4_@SiO_2_ using the co-precipitation method^36,37^. To introduce l-lysine functional groups onto the Fe_3_O_4_@SiO_2_ surface, 1 g of the synthesized Fe_3_O_4_@SiO_2_ was suspended in 30 mL of ethanol solution. Gradually, 1.5 mmol or 0.219 g of l-lysine was added to this solution while ensuring continuous mixing. The resulting mixture underwent stirring under reflux conditions over a duration of 24 h to achieve functionalization. Following this, the solid material was carefully separated through filtration, washed multiple times with ethanol to remove impurities, and subsequently air-dried at ambient temperature. To synthesize Fe_3_O_4_@SiO_2_-LY-C, 1 g of the l-lysine-functionalized Fe_3_O_4_@SiO_2_ (denoted as Fe_3_O_4_@SiO_2_-LY) was dispersed uniformly in 40 mL of tetrahydrofuran (THF) via sonication for 30 min to ensure homogeneity. Afterward, 2.5 mmol or 0.461 g of cyanuric chloride was added to the reaction vessel, and the reaction mixture was stirred at room temperature for 24 h to facilitate covalent attachment. Upon completion of the reaction, the resulting Fe_3_O_4_@SiO_2_-LY-C product was isolated using magnetic separation. The solid material was then thoroughly washed three times with fresh THF to remove any unreacted reagents and by-products. Following this purification step, the material was dried in an oven set at 40 °C for 5 h. To further modify this intermediate product, the dried Fe_3_O_4_@SiO_2_-LY-C sample was treated with a mixture of 40 mL of acetonitrile and 1 mL of diisopropylethylamine. Subsequently, 7 mmol (equivalent to 1.2 g) of dipyridylamine was introduced into the reaction mixture. This suspension was initially stirred at room temperature for 2 h to ensure proper interaction between the reactants and then subjected to reflux conditions for an extended period of 20 h to allow the reaction to proceed to completion. Afterward, the solid products were separated using an external magnet, followed by thorough washing with deionized water and acetone to remove residual reactants. Finally, the solid product was oven-dried at a controlled temperature of 50 °C for 12 h. To synthesize the final Fe_3_O_4_@SiO_2_-LY-C-D-Pd nanocatalyst, a mixture containing 1 g of the Fe_3_O_4_@SiO_2_-LY-C-D precursor, 2.5 mmol of palladium acetate (Pd(OAc)_2_), and 40 mL of ethanol was prepared and added to a reaction vessel. This mixture was stirred under reflux conditions for 24 h to facilitate palladium loading onto the nanostructure. Subsequently, 1.6 mmol of sodium borohydride (NaBH_4_) was gradually introduced into the flask to promote the reduction of palladium ions to metallic palladium particles. This reduction process was carried out with agitation over a period of 6 h. Once complete, the resulting nanocatalyst was separated using magnetic extraction and subjected to multiple rinse cycles with ethanol and water to ensure thorough purification. Finally, the nanocatalyst was dried under vacuum conditions at 50 °C, producing the final product ready for catalytic applications (Fig. 1).

### General procedure for the Suzuki reactions

**Fig. 2:**
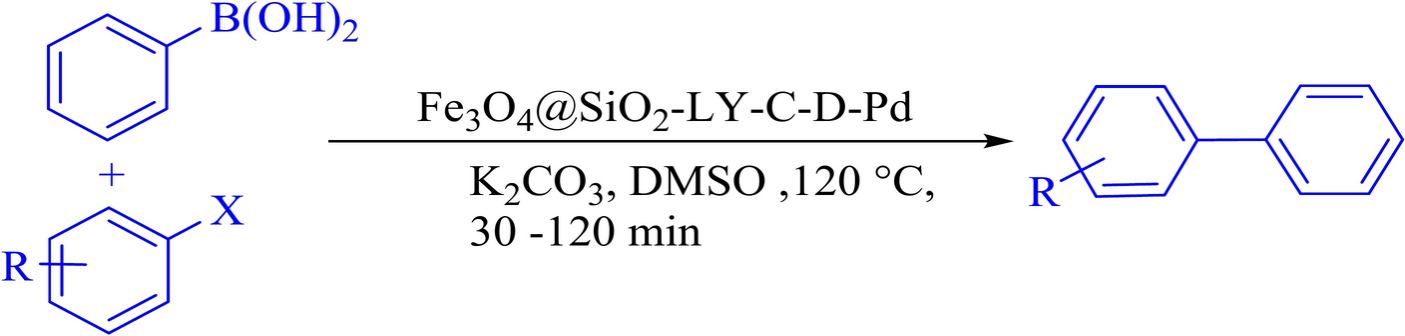
Fe_3_O_4_@SiO_2_-LY-C-D-Pd‐catalysed Suzuki reaction.

In a 25 mL round bottom flask, aryl halide (1.0 mmol), phenylboronic acid (1.0 mmol or 0.129 g), K_2_CO_3_ (1.5 mmol or 0.207 g), and Fe_3_O_4_@SiO_2_-LY-C-D-Pd (30 mg) were added to DMSO (4 mL) and stirred at 120 °C for 30 min. After the reaction was finished (monitored via thin-layer chromatography), 10 mL of EtOAc was added to the reaction mixture, and the nanocatalyst was separated using a magnet. The organic phase yielded biphenyl compounds through solvent evaporation (Fig. 2).

### General procedure for synthesis of 1H‐tetrazoles

**Fig. 3:**
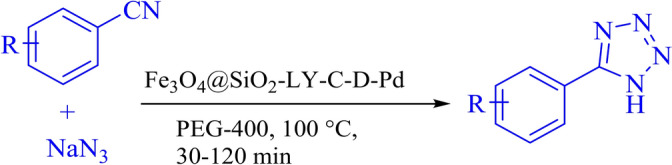
Synthesis of ^1^H-tetrazoles in the presence of Fe_3_O_4_@SiO_2_-LY-C-D-Pd.

A reaction involved combining 1.3 mmol or 0.084 g of sodium azide, 1 mmol of nitrile, and 0.03 g of Fe_3_O_4_@SiO_2_-LY-C-D-Pd in PEG-400 (2 mL) and stirring the mixture at 120 °C. After completion, determined by TLC monitoring, the mixture was allowed to cool to room temperature. The catalyst was separated using a magnet, and HCl (4 N, 2 mL) was added to the filtered solution. The resulting tetrazole was extracted with ethyl acetate, and the organic phase was washed with distilled water, dried with anhydrous sodium sulfate, and concentrated to yield the crude solid product (Fig. 3).

### Catalyst characterizations

**Fig. 4:**
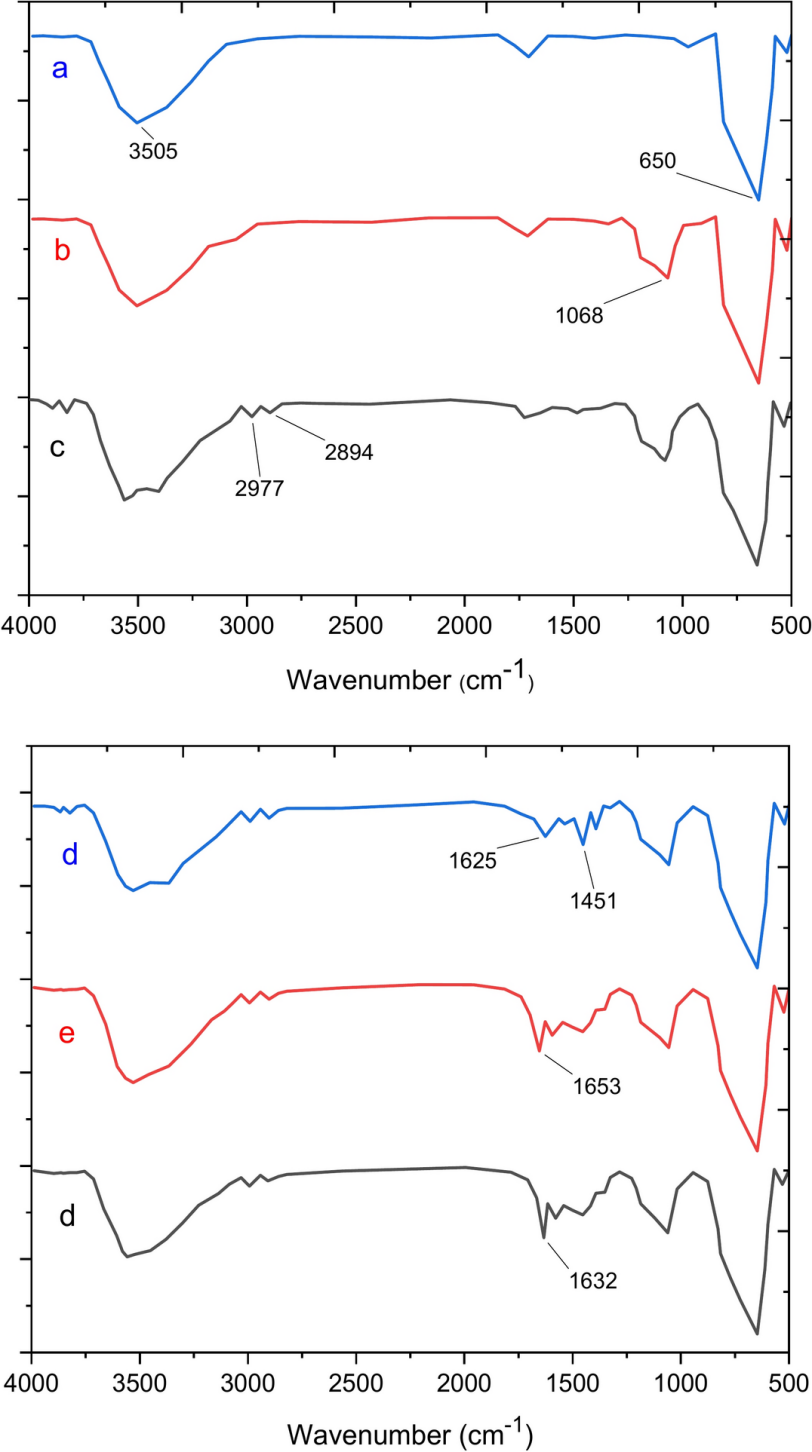
Comparative study of FTIR spectra of (**a**) Fe_3_O_4_, (**b**) Fe_3_O_4_@SiO_2_, (**c**) Fe_3_O_4_@SiO_2_-LY (**d**) Fe_3_O_4_@SiO_2_-LY@C, (**e**) Fe_3_O_4_@SiO_2_-LY-C-D (**f**) Fe_3_O_4_@SiO_2_-LY-C-D-Pd.

FT-IR analysis (Fig. 4) shows the FT-IR spectra of Fe_3_O_4_, Fe_3_O_4_@SiO_2,_ Fe_3_O_4_@SiO_2_-LY, Fe_3_O_4_@SiO_2_-LY-C, Fe_3_O_4_@SiO_2_-LY-C-D and Fe_3_O_4_@SiO_2_-LY-C-D-Pd MNPs. The two absorption bands at 650 cm^−1^ are attributed to the stretching vibrations of iron-oxygen bonds. In Fig. 1a, the bending and stretching vibrations of OH molecules present on the nanoparticle surface are observed at 3435 cm^−1^. Figure 4b illustrates the occurrence of a condensation reaction between the hydroxyl groups of Fe_3_O_4_ nanoparticles (MNPs) and the alkoxysilane molecules of TEOS, forming the first layer. The peak at 1068 cm^−1^ confirms the presence of Si–O–Si stretching vibrations, which correspond to the silica shell. In Fig. 4c, for Fe_3_O_4_@SiO_2_-LY, the bands observed at 2889 and 2809 cm^−1^ correspond to the bending vibration of CH_2_, confirming the successful attachment of l-lysine chain molecules. In Fig. 4d, the emergence of new bands at 1458 and 1561 cm^−1^ (from the Fe_3_O_4_@SiO_2_-LY@C spectrum) signifies the presence of an aromatic triazine ring in the Fe_3_O_4_@SiO_2_-LY-C sample, confirming the stabilization of cyanuric chloride. The incorporation of pyridine groups is validated through bands appearing at 3050 cm^−1^ (aromatic C-H stretching) and 1653 cm^−1^ (C-N stretching). These results indicate that Fe_3_O_4_@SiO_2_ has been successfully functionalized with melamine-containing pyridine groups, as shown in Fig. 4e. Additionally, the peak at 1653 cm^−1^ in Fig. 4e signifies metal–ligand coordination, with its shift to a lower frequency range (1653–1632 cm^−1^) further confirming the successful binding of palladium ions (Pd) with the organic ligand, as depicted in Fig. 4f^37,38^.

**Fig. 5:**
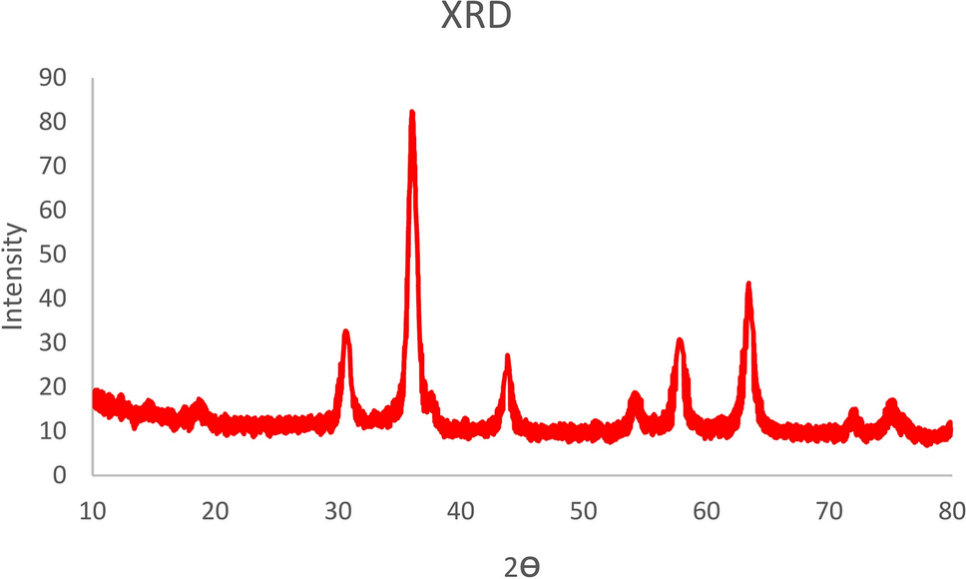
XRD spectrum of Fe_3_O_4_@SiO_2_-LY-C-D-Pd.

Figure 5 illustrates the normal-angle powder X-ray diffraction (XRD) patterns of Fe_3_O_4_@SiO_2_-LY-C-D-Pd. The XRD pattern closely resembles that of Fe_3_O_4_, confirming the presence of octahedral structures. The successful synthesis of Fe_3_O_4_ nanoparticles (MNPs) is evident from the peak positions observed at 2θ = 31°, 37°, 44°, 56°, 61°, and 68°, which correspond to the (2 2 0), (3 1 1), (4 0 0), (4 2 2), (5 1 1), and (4 4 0) crystal plane reflections, respectively. Moreover, the XRD data confirm a cubic spinel crystalline structure characteristic of Fe_3_O_4_. These results suggest that the structural integrity of Fe_3_O_4_ was retained during the preparation of the LY-C-D-Pd-supported catalyst, with its crystalline phase and structural characteristics remaining largely unaltered.

**Fig. 6:**
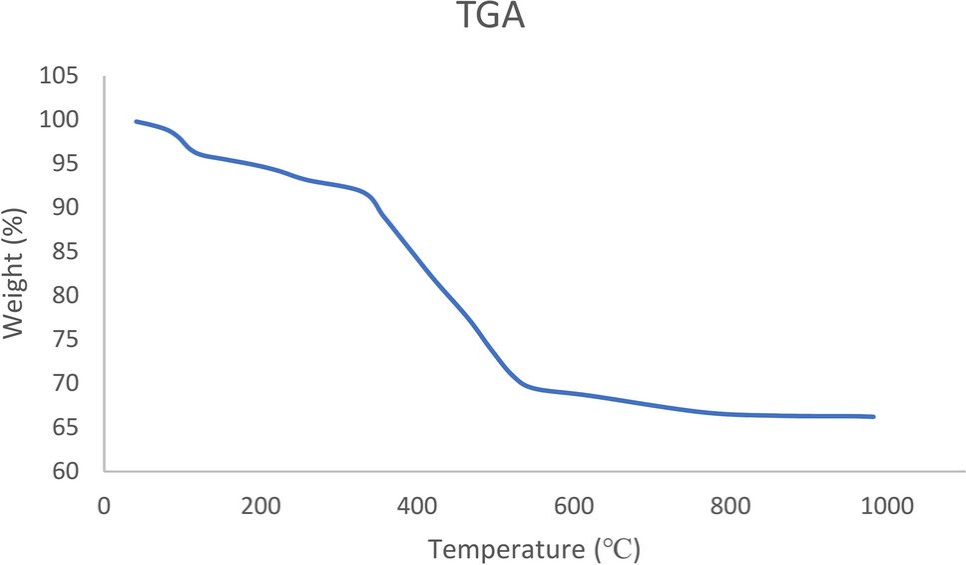
TGA curve of Fe_3_O_4_@SiO_2_-LY-C-D-Pd.

The thermal behavior of the Fe_3_O_4_@SiO_2_-LY-C-D-Pd complex nanocomposite was analyzed using TGA, as illustrated in Fig. 6. The analysis revealed two distinct weight loss regions. The first, approximately 5%, occurred below 250 °C and was attributed to the removal of organic solvents trapped within the catalyst structure. The second weight loss, around 26%, was observed between 250 and 600 °C, corresponding to the decomposition of the organic layer and the Pd complex attached to Fe_3_O_4_ (Fig. 6). These findings confirmed the successful chemisorption of the LY-C-D-Pd complex onto the surface of Fe_3_O_4_@SiO_2_ MNPs.

**Fig. 7:**
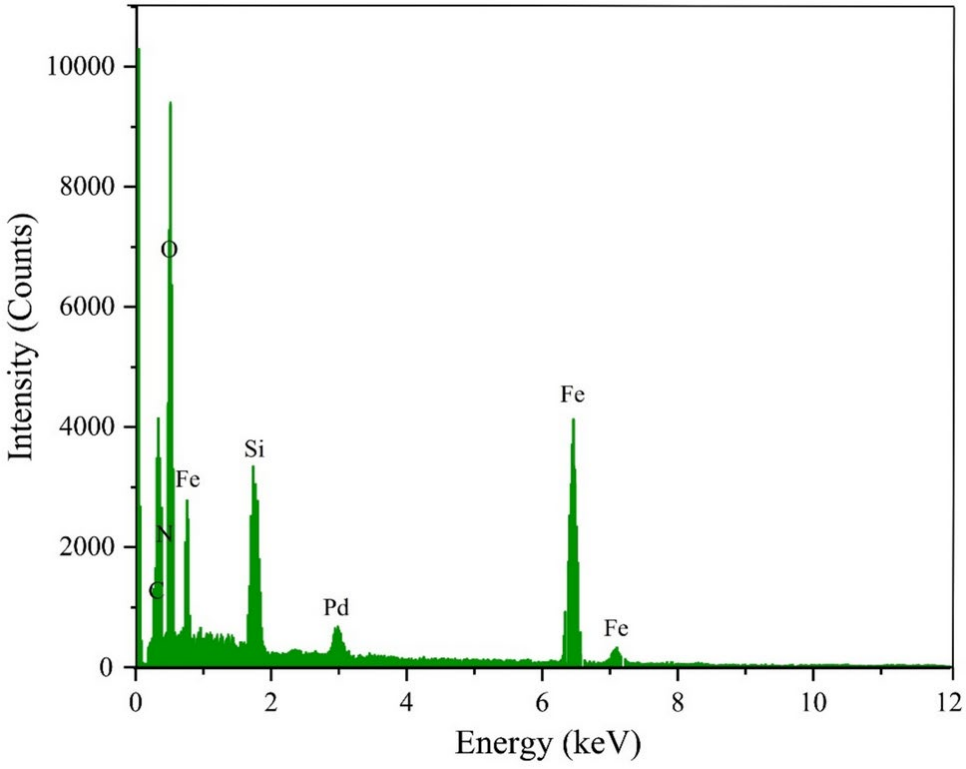
EDS analysis of Fe_3_O_4_@SiO_2_-LY-C-D-Pd.

The elemental composition of Fe_3_O_4_@SiO_2_-LY-C-D-Pd was determined from the EDX spectrum (Fig. 7). Figure 7 confirms the presence of Silicon, Iron, Nitrogen, Oxygen, Carbon, and Palladium in the catalyst and proves the successful synthesis of nanoparticles. The results confirmed the successful immobilization of LY-C-D-Pd on the surface of Fe_3_O_4_@SiO_2_ MNPs.

**Fig. 8:**
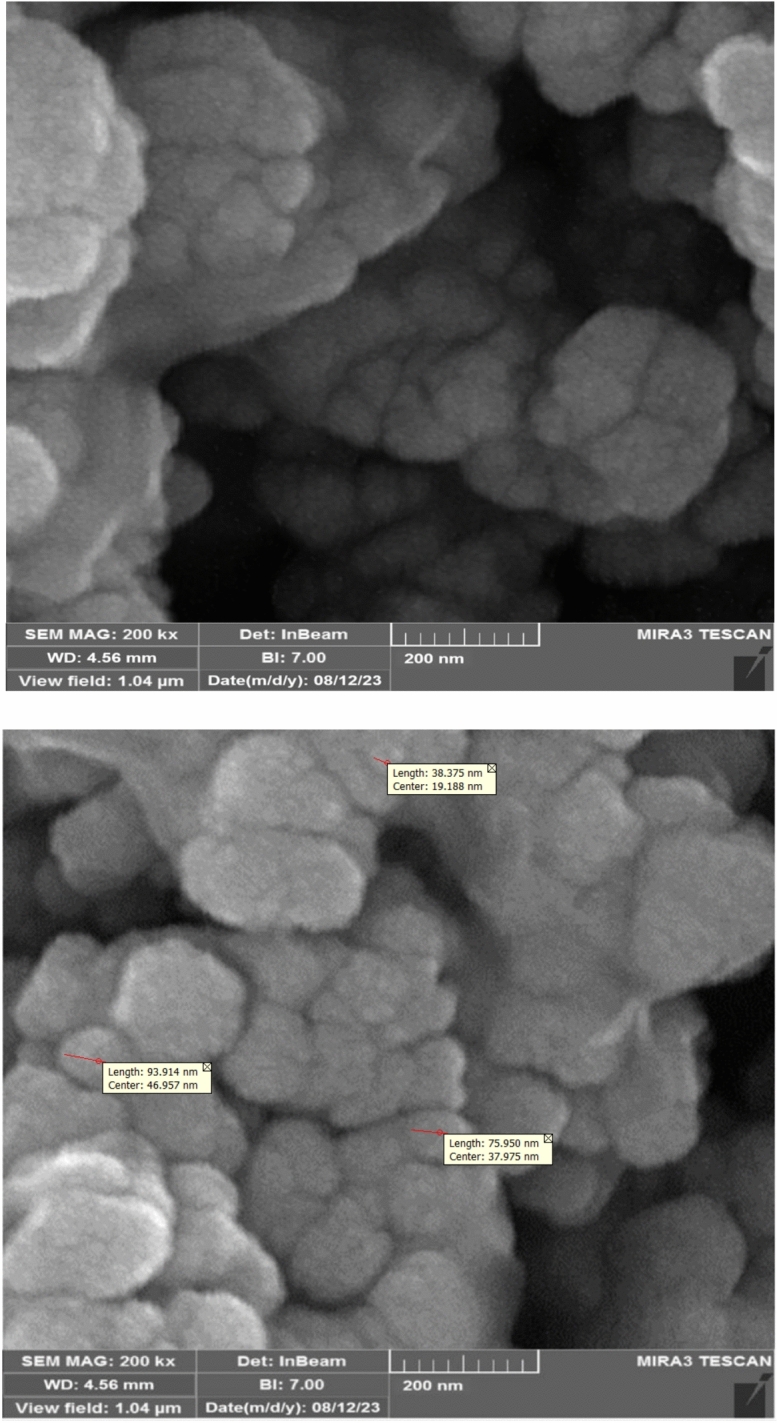
SEM images of Fe_3_O_4_@SiO_2_-LY-C-D-Pd.

The size and morphology of the synthesized Fe_3_O_4_@SiO_2_-LY-C-D-Pd particles were analyzed using scanning electron microscopy (SEM). The SEM image revealed the formation of uniform, single-dispersed nanoparticles. A closer examination of the magnified image indicated slight aggregation and stacking textures, likely caused by magnetic interactions within the catalyst’s particle structure (Fig. 8). The average particle size was measured to range between 38 and 93 nm.

**Fig. 9:**
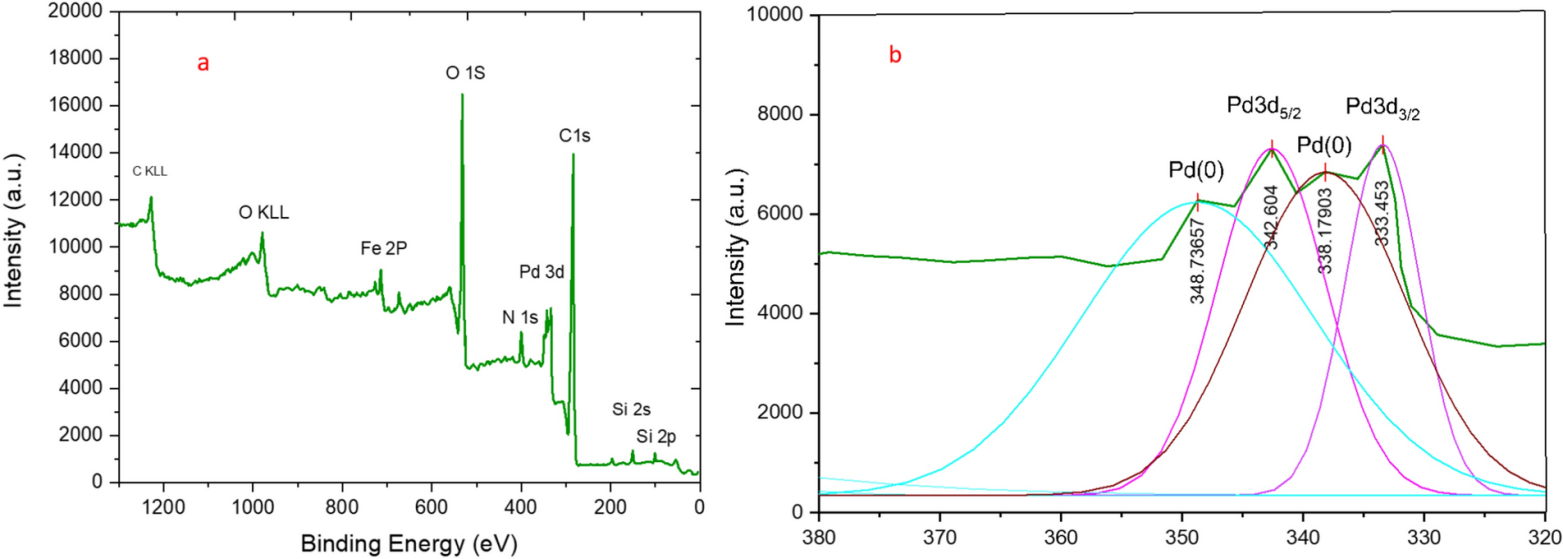
XPS spectrum of Fe_3_O_4_@SiO_2_-LY-C-D-Pd catalyst.

Figure 9 presents the XPS spectrum of the synthesized Fe_3_O_4_@SiO_2_-LY-C-D-Pd catalyst, showcasing peaks corresponding to oxygen, carbon, silicon, nitrogen, palladium, and iron (Fig. 9a). The oxidation state of palladium was examined through XPS analysis, as shown in Fig. 9b. To determine this oxidation state, X-ray photoelectron spectroscopy (XPS) studies were conducted, revealing two distinct binding energy peaks at 333.4 eV and 342.6 eV. These peaks are attributed to Pd 3d_3/2_ and Pd 3d_5/2_, respectively. Based on this analysis, the XPS data confirms the structure of the synthesized Fe_3_O_4_@SiO_2_-LY-C-D-Pd catalyst.

**Fig. 10:**
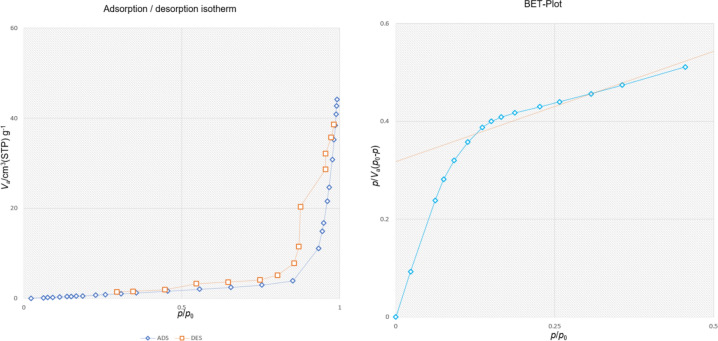
N_2_-adsorption isotherms of Fe_3_O_4_@SiO_2_-LY-C-D-Pd.

The pore size and surface area distribution of Fe_3_O_4_@SiO_2_-LY-C-D-Pd were carefully examined using the N_2_ adsorption–desorption isotherms technique, as illustrated in Fig. 10. This method revealed that the surface area of Fe_3_O_4_@SiO_2_-LY-C-D-Pd is 6.15 m^2^/g. Furthermore, the Barrett–Joyner–Halenda (BJH) method provided details about the pore size distribution and volume, showing dimensions of 40.56 nm for pore size and a pore volume of 0.06 cm^3^/g.

In addition, an ICP-OES analysis was conducted to determine the quantity of Pd in Fe_3_O_4_@SiO_2_-LY-C-D-Pd. According to the analysis, the Pd content in the catalyst was measured at 1.5 × 10^–4^ mol g^−1^. Furthermore, the extent of Pd leaching following the catalyst’s recycling was examined through ICP analysis. The findings reveal that the levels of Pd in the reused catalysts are 1.4 × 10^–4^ mol g^−1^, indicating minimal Pd leaching from the Fe_3_O_4_@SiO_2_-LY-C-D-Pd framework.

**Fig. 11:**
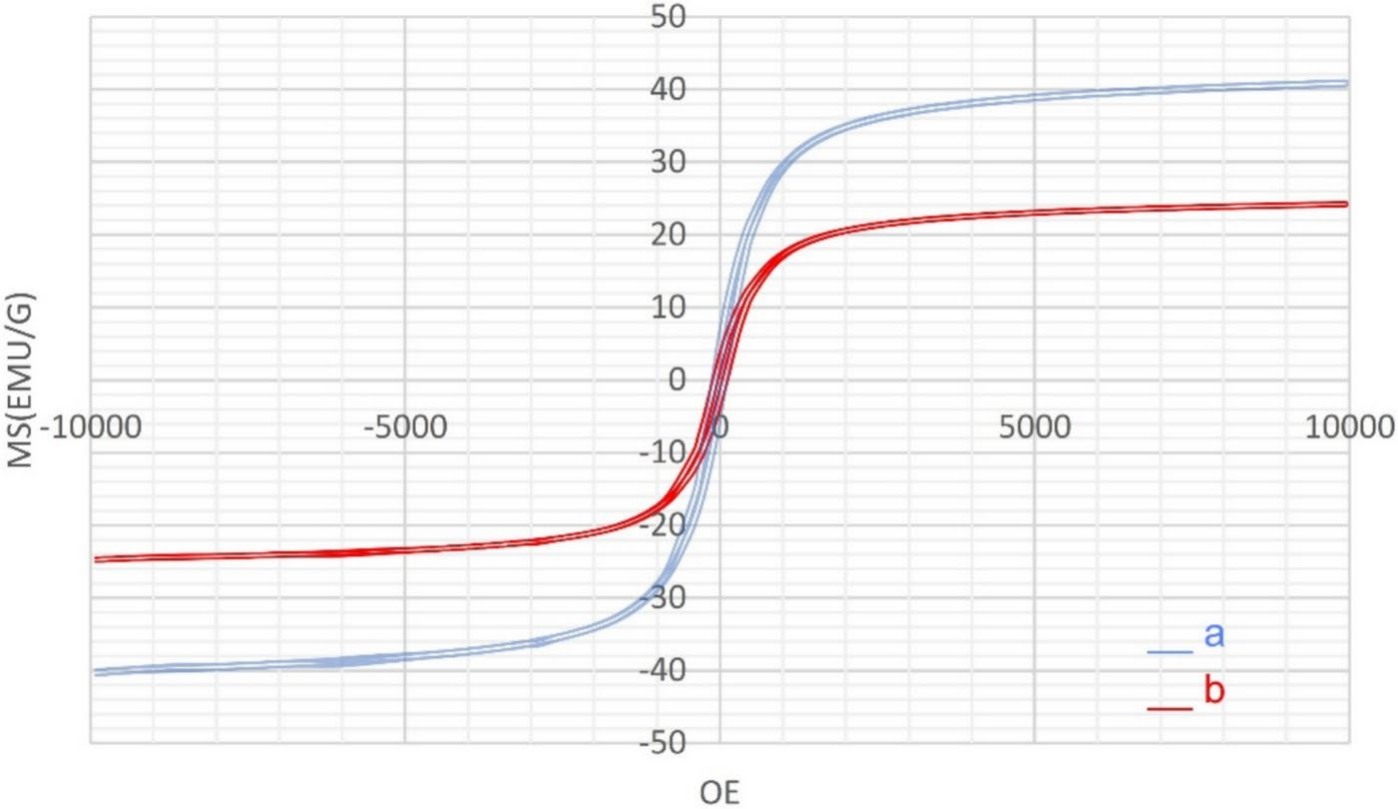
VSM curves of (**a**) Fe_3_O_4_ (**b**) Fe_3_O_4_@SiO_2_-LY-C-D-Pd.

The magnetic properties of uncoated magnetic spinel ferrite (Fe_3_O_4_) and Fe_3_O_4_@SiO_2_-LY-C-D-Pd MNPs were examined through VSM analysis within the external magnetic field range of − 10,000 to + 10,000 Oe at room temperature (see Fig. 11). As shown in Fig. 11, the decrease in saturation magnetization from approximately 40 emu/g to about 29 emu/g can be attributed to the presence of the new coated layer, serving as evidence of the successful synthesis of the catalyst. Though the Ms level of the Fe_3_O_4_@SiO_2_-LY-C-D-Pd is lower than that of the Fe_3_O_4_ NPs its magnetic sensitivity is sufficient for its magnetic removal from different reaction mediums.

### Catalytic studies

The Fe_3_O_4_@SiO_2_-LY-C-D-Pd catalyst was studied for its efficiency in synthesizing biaryls through the Suzuki cross-coupling reaction. Iodobenzene and PhB(OH)_2_ were selected as the model reactants for this investigation. Reaction conditions were optimized by varying the amount of catalyst, solvent, temperature, and other parameters. According to Table 1, no reaction occurred in the absence of the catalyst, even after an extended period (entry 1). Using 8 mg of the catalyst resulted in a low product yield (entry 2). However, when 30 mg of the catalyst was employed with suitable proportions of aryl halide and PhB(OH)_2_, optimal performance was achieved. Increasing the temperature to 120 °C resulted in a high product yield within just 30 min. Further optimization involved testing different bases, such as K_2_CO_3_, KOH, and NaOH. Among them, K_2_CO_3_ proved most effective, providing a 98% yield of the desired biphenyl product within 30 min (entry 5). The solvent selection was also explored using options like DMSO, EtOH, DMF, PEG-400, CH_3_CN, water, and a solvent-free system (entries 7–13). The use of DMSO stood out, delivering a 98% yield of biphenyl after 30 min.

**Table 1:** Optimization of the reaction conditions for the coupling reaction of phenylboronic acid with iodobenzene.


Entry	Catalyst	Amount (mg)	Solvent	Base	Temperature (°C)	Time (min)	Yield (%)
1	Fe_3_O_4_@SiO_2_-LY-C-D-Pd	-	DMSO	K_2_CO_3_	120	1 day	N. R
2	Fe_3_O_4_@SiO_2_-LY-C-D-Pd	8	DMSO	K_2_CO_3_	120	30	37
3	Fe_3_O_4_@SiO_2_-LY-C-D-Pd	10	DMSO	K_2_CO_3_	120	30	73
4	Fe_3_O_4_@SiO_2_-LY-C-D-Pd	15	DMSO	K_2_CO_3_	120	30	89
5	Fe_3_O_4_@SiO_2_-LY-C-D-Pd	30	DMSO	K_2_CO_3_	120	30	98
6	Fe_3_O_4_@SiO_2_-LY-C-D-Pd	35	DMSO	K_2_CO_3_	120	30	98
7	Fe_3_O_4_@SiO_2_-LY-C-D-Pd	30	EtOH	K_2_CO_3_	Reflux	30	45
8	Fe_3_O_4_@SiO_2_-LY-C-D-Pd	30	PEG-400	K_2_CO_3_	120	30	56
9	Fe_3_O_4_@SiO_2_-LY-C-D-Pd	30	H_2_O	K_2_CO_3_	Reflux	30	83
10	Fe_3_O_4_@SiO_2_-LY-C-D-Pd	30	Solvent-free	K_2_CO_3_	120	30	81
11	Fe_3_O_4_@SiO_2_-LY-C-D-Pd	30	MeOH	K_2_CO_3_	Reflux	30	91
12	Fe_3_O_4_@SiO_2_-LY-C-D-Pd	30	CH_3_CN	K_2_CO_3_	Reflux	30	Trac
13	Fe_3_O_4_@SiO_2_-LY-C-D-Pd	30	DMF	K_2_CO_3_	120	30	–
14	Fe_3_O_4_@SiO_2_-LY-C-D-Pd	30	DMSO	K_2_CO_3_	25	30	44
15	Fe_3_O_4_@SiO_2_-LY-C-D-Pd	30	DMSO	K_2_CO_3_	60	30	60
16	Fe_3_O_4_@SiO_2_-LY-C-D-Pd	30	DMSO	K_2_CO_3_	80	30	83
17	Fe_3_O_4_@SiO_2_-LY-C-D-Pd	30	DMSO	KOH	120	30	81
18	Fe_3_O_4_@SiO_2_-LY-C-D-Pd	30	DMSO	NaOH	120	30	76
19	Fe_3_O_4_@SiO_2_-LY-C-D	30	DMSO	K_2_CO_3_	120	30	N. R

After achieving the best conditions for coupling iodobenzene with phenylboronic acid, the catalytic activity of Fe_3_O_4_@SiO_2_-LY-C-D-Pd was expanded to include the coupling of other aryl halides with phenylboronic acid. Furthermore, a variety of aryl bromides, iodides, and chlorides were examined in the coupling reaction with phenylboronic acid using Fe_3_O_4_@SiO_2_-LY-C-D-Pd (Table 2). In this case, the Suzuki–Miyaura reaction successfully yielded the desired biphenyl derivatives from both electron-deficient and electron-rich aryl halides under mild conditions.

**Table 2:**
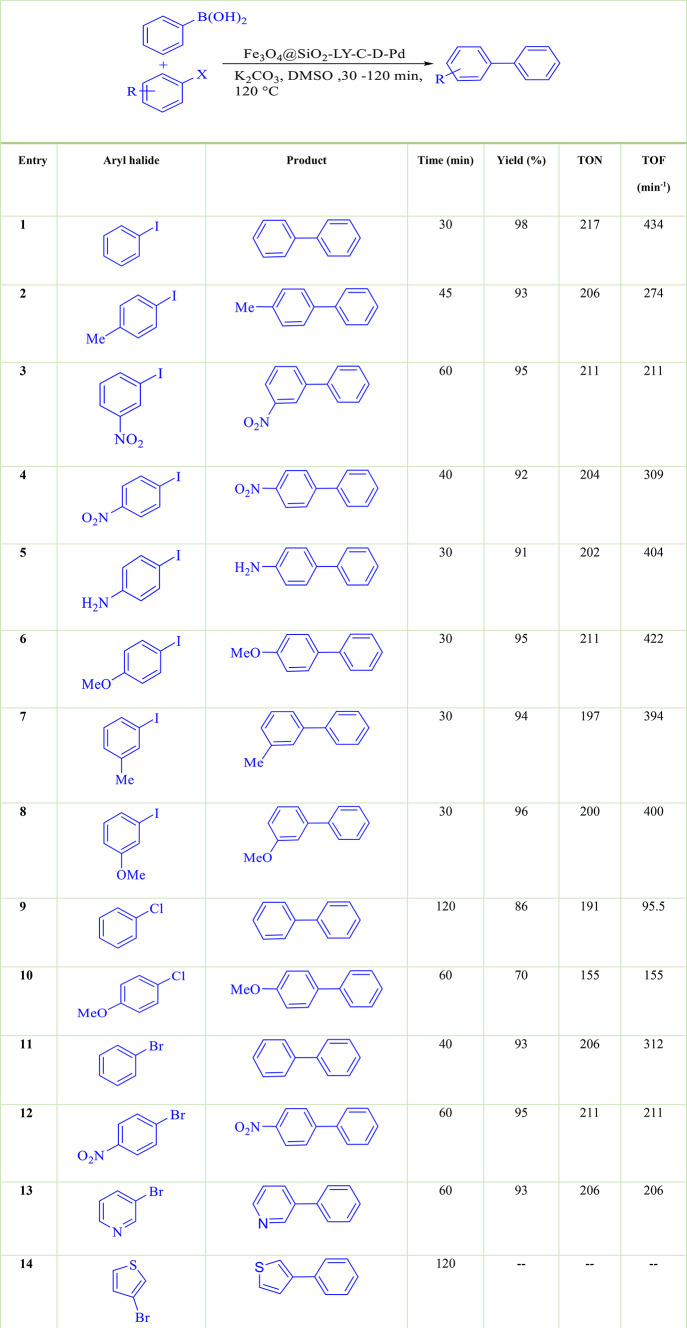
Catalytic coupling reaction of various aryl halides with phenylboronic acid.

**Fig. 12:**
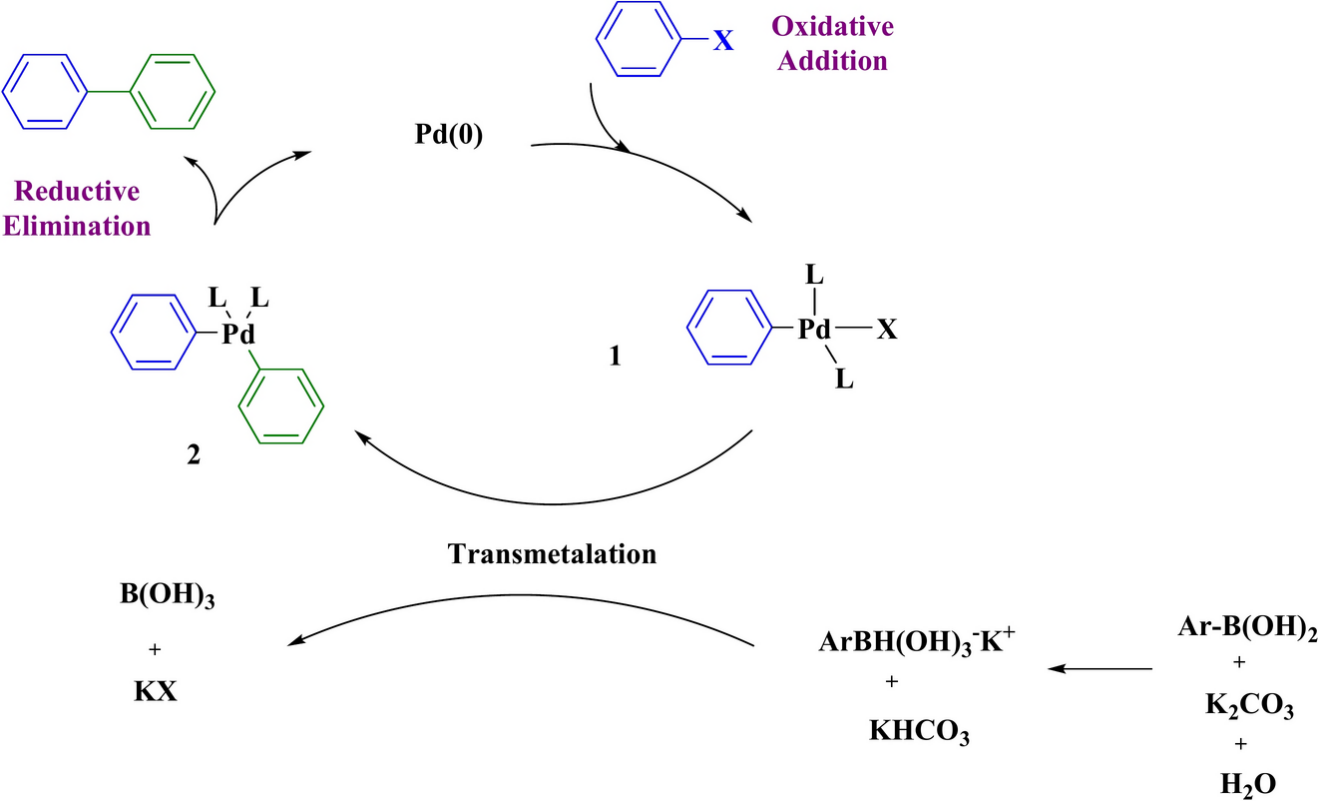
Possible mechanism for Suzuki reaction.

Predicting the precise course of a chemical reaction in its entirety can be challenging. However, here we propose a mechanism for the synthesis involved in the Suzuki reaction. In this coupling mechanism, aryl halide undergoes an oxidative addition with Pd(0). Subsequently, the base potassium carbonate activates phenylboronic acid, leading to the formation of a phenyl boronate ester. Next, medium (2) forms through a displacement reaction, where the metal in medium (1) is substituted by the activated borane group. The final step involves an elimination-reduction process, yielding the desired product while regenerating the catalyst for reuse (Fig. 12).

We next focused on the cycloaddition reaction of aryl benzonitriles with sodium azide. To identify the optimal conditions, a representative reaction between benzonitrile and NaN_3_ was selected in a 1:1.3 molar ratio, with varying parameters such as solvent, temperature, and catalyst loading using a Pd-anchored catalyst. The findings are summarized in Table 3. The study evaluated key factors, including temperature, different solvents (such as PEG-400, n-Hexane, H_2_O, EtOH, CH_3_CN, and a solvent-free method), the amount of catalyst, and the quantity of NaN_3_ in the reaction model. From the results detailed in Table 3, it was concluded that the optimal conditions for synthesizing 1H-tetrazole derivatives involve using 0.03 g of Fe_3_O_4_@SiO_2_-LY-C-D-Pd in PEG-400 at 120 °C with 1.3 mmol of NaN_3_.

**Table 3:** Optimization of reaction conditions for the synthesis of 1H‐tetrazole derivatives.


Entry	Catalyst	Amount (mg)	Solvent	NaN_3_(mmol)	Temperature(°C)	Time(min)	Yield (%) ^a^
1	Fe_3_O_4_@SiO_2_-LY-C-D-Pd	-	PEG	1.3	120	24 h	NR
2	Fe_3_O_4_@SiO_2_-LY-C-D-Pd	10	PEG	1.3	120	45	48
3	Fe_3_O_4_@SiO_2_-LY-C-D-Pd	20	PEG	1.3	120	45	85
4	Fe_3_O_4_@SiO_2_-LY-C-D-Pd	30	PEG	1.3	120	45	98
5	Fe_3_O_4_@SiO_2_-LY-C-D-Pd	40	PEG	1.3	120	45	98
6	Fe_3_O_4_@SiO_2_-LY-C-D-Pd	30	EtOH	1.3	Reflux	45	67
7	Fe_3_O_4_@SiO_2_-LY-C-D-Pd	30	H_2_O	1.3	Reflux	45	65
8	Fe_3_O_4_@SiO_2_-LY-C-D-Pd	30	Solvent-free	1.3	120	45	59
9	Fe_3_O_4_@SiO_2_-LY-C-D-Pd	30	CH_3_CN	1.3	Reflux	45	43
10	Fe_3_O_4_@SiO_2_-LY-C-D-Pd	30	n-Hexane	1.3	Reflux	45	Trac
11	Fe_3_O_4_@SiO_2_-LY-C-D-Pd	30	PEG	1.3	25	45	N. R
12	Fe_3_O_4_@SiO_2_-LY-C-D-Pd	30	PEG	1.3	50	45	40
13	Fe_3_O_4_@SiO_2_-LY-C-D-Pd	30	PEG	1.3	80	45	51
14	Fe_3_O_4_@SiO_2_-LY-C-D-Pd	30	PEG	1	120	45	92
15	Fe_3_O_4_@SiO_2_-LY-C-D-Pd	30	PEG	0.7	120	45	74

^a^Isolated yield.

After fine-tuning the reaction conditions, the catalytic scope of Fe_3_O_4_@SiO_2_-LY-C-D-Pd was broadened to encompass both aromatic and aliphatic nitrile derivatives. The study particularly concentrated on aromatic nitriles with either electron-withdrawing or electron-donating groups on the aromatic ring to produce corresponding tetrazole derivatives. As shown in Table 4, all products were synthesized within acceptable durations and attained outstanding yields, showcasing the catalyst’s exceptional efficiency. Moreover, para-, meta-, and ortho-substituted benzonitriles were effectively examined. The investigation also extended to aliphatic nitriles, resulting in tetrazole derivatives with excellent yields. An interesting property observed was homoselectivity, where only one similar functional group participates in the reaction. For instance, the catalyst was used to study malononitrile, which contains two identical cyano groups, for tetrazole production. Impressively, Fe_3_O_4_@SiO_2_-LY-C-D-Pd demonstrated high selectivity by reacting with only one cyano group in malononitrile with sodium azide while leaving the other unchanged.

**Table 4:**
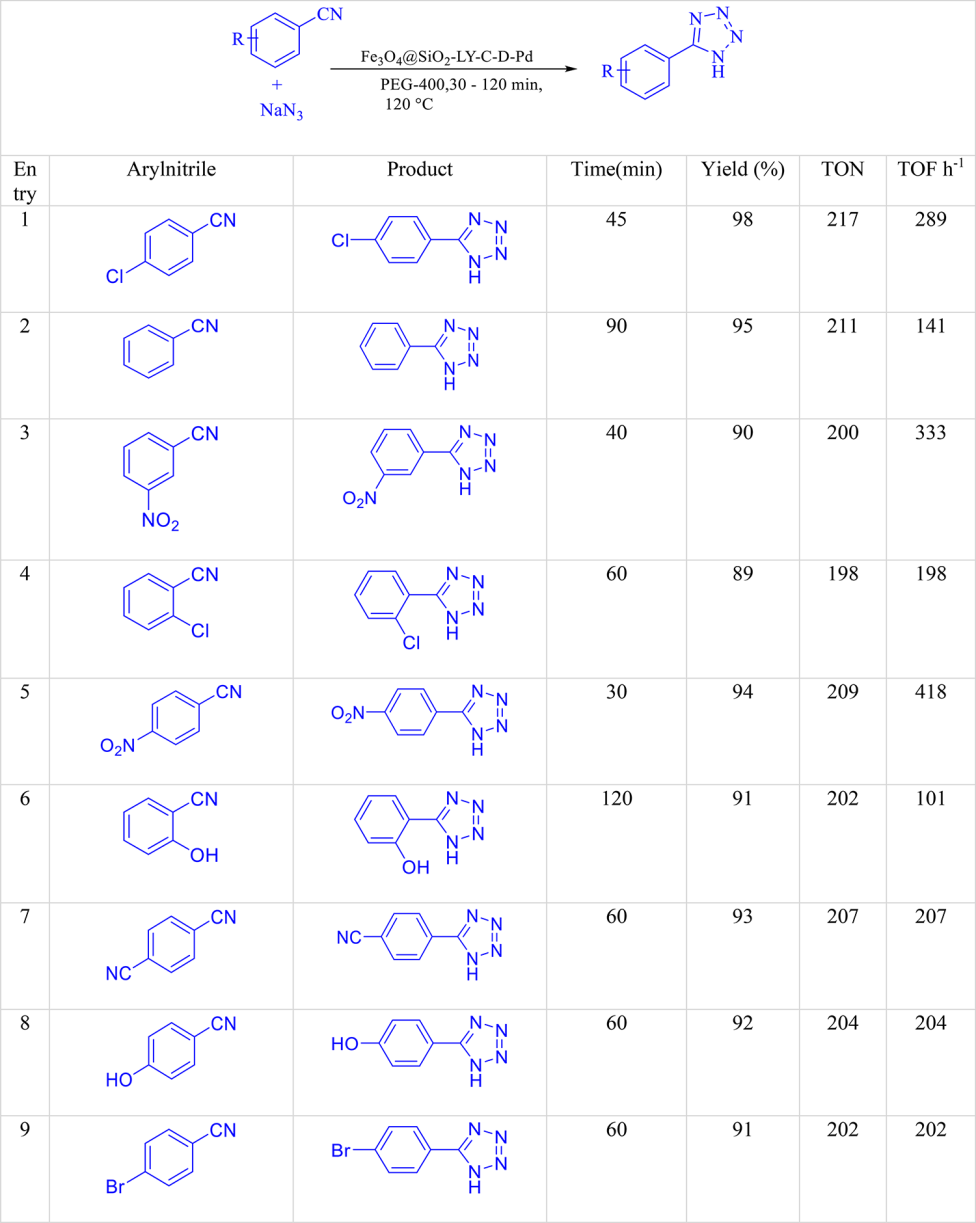
Synthesis of different structurally 1H-tetrazoles catalyzed by Fe_3_O_4_@SiO_2_-LY-C-D-Pd.

**Fig. 13:**
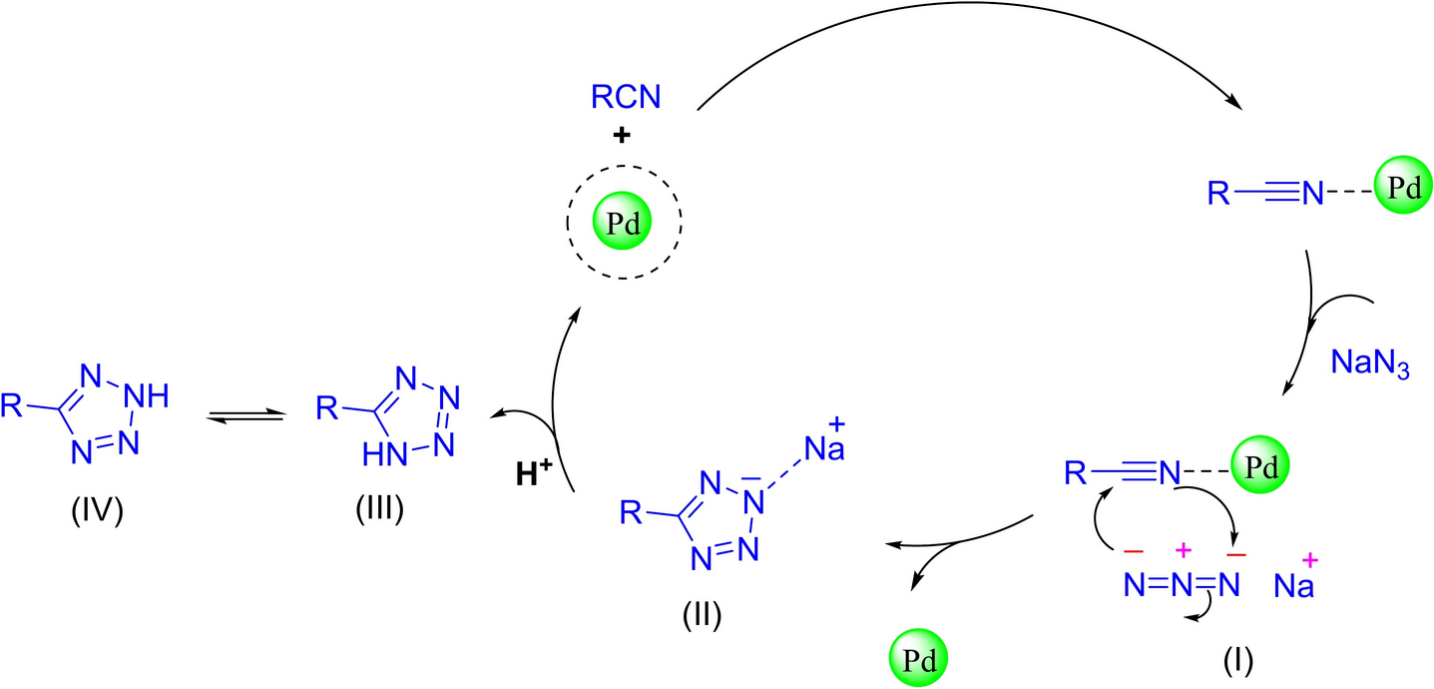
Proposed Mechanism for tetrazole in the presence of Fe_3_O_4_@SiO_2_-LY-C-D-Pd.

Previous research has outlined an efficient cyclic mechanism for synthesizing 5-substituted 1H-tetrazoles using the Fe_3_O_4_@SiO_2_-LY-C-D-Pd catalyst, as depicted in Fig. 13. In this process, the cyanide functional group is initially activated by palladium metal, enabling its reaction with sodium azide to yield intermediate I. Subsequently, the removal of the catalyst facilitates the formation of intermediate II. In the final step, intermediate II is transformed into the desired tetrazole product through the addition of HCl. Concurrently, the catalyst is regenerated, making it ready to initiate a new reaction cycle.

## Catalyst recyclability

**Fig. 14:**
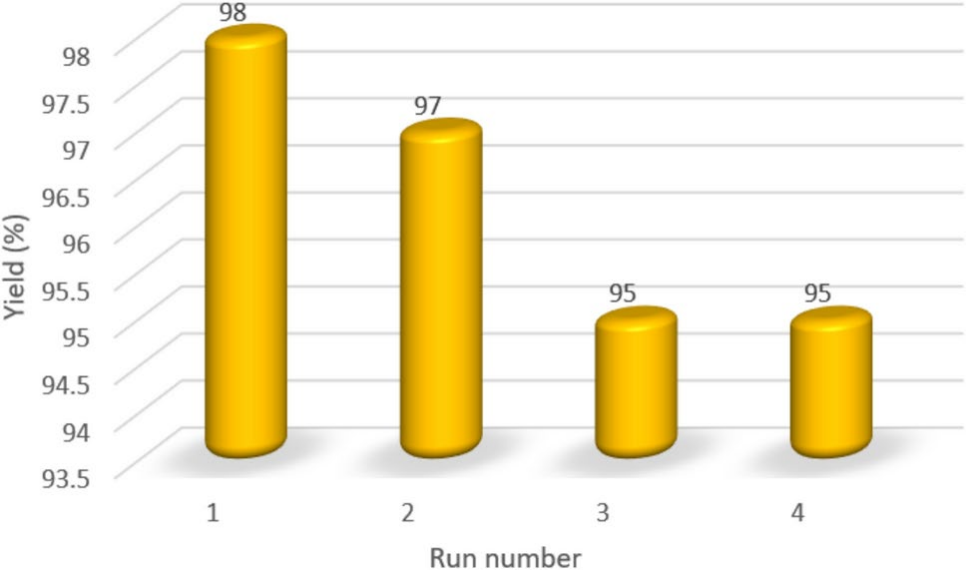
Recyclability of Fe_3_O_4_@SiO_2_-LY-C-D-Pd in the synthesis of Suzuki reaction.

The Fe_3_O_4_@SiO_2_-LY-C-D-Pd was tested for its recyclability in the production of biphenyl under the best possible conditions. The Fe_3_O_4_@SiO_2_-LY-C-D-Pd was reused up to four catalytic cycles without noticeable change in activity (Fig. 14).

The efficiency of Fe_3_O_4_@SiO_2_-LY-C-D-Pd as catalyst was considered by comparing these obtained results with previously reported catalysts in the authentic literature (Table 5). Therefore, it can be seen that Fe_3_O_4_@SiO_2_-LY-C-D-Pd is an efficient and useful catalyst for the synthesis of biphenyl compounds.

**Table 5:** Comparing catalytic activity of Fe_3_O_4_@SiO_2_-LY-C-D-Pd with previously reported methods in the C–C coupling reaction.

Entry	Catalyst	Product	Time (mine)	Yield (%)	Refs.
1	Fe_3_O_4_@chamomile-Pd	1,1′-biphenyl	1 h	96	^39^
2	Fe_3_O_4_@SiO_2_@ Am-Pd	1,1′-biphenyl	1 h	96	^40^
3	Fe_3_O_4_@SiO_2_-Pd	1,1′-biphenyl	30	95	^41^
4	Fe_3_O_4_@SiO_2_-LY-C-D-Pd	1,1′-biphenyl	30	98	This work

## Conclusion

In this work, Fe_3_O_4_@SiO_2_-LY-C-D-Pd as a bimetallic heterogeneous nanocatalyst was successfully synthesized and characterized by different techniques such as FT-IR, TGA, EDS, SEM, XRD, ICP, and VSM study. Fe_3_O_4_@SiO_2_-LY-C-D-Pd nanocomposite catalyzed the C–C coupling reaction and ^1^H-tetrazoles with high yields in short reaction times. Modifying the Fe_3_O_4_@SiO_2_-LY nanoparticles with C-D-Pd significantly enhanced catalytic efficiency, resulting in exceptional yields of the reaction products. The catalyst’s sustainability has been proven by its ability to be reused for multiple reaction cycles without any significant decrease in its effectiveness. Furthermore, this new Fe_3_O_4_@SiO_2_-LY-C-D-Pd can be readily prepared from commercially available materials. Simple and environmentally benign procedures, clean reaction profiles, short reaction times, excellent yields, low catalyst loading, and good chemical stability of nanocatalysts are the considerable features of this method.

## Supplementary Information

Supplementary Information.

## Data Availability

All data generated or analyzed during this study are included in this published article and its supplementary information files.
