# Supplementary material for: Fe3O4@SiO2-LY-C-D-Pd as a new, effective, and magnetically recoverable catalyst for the synthesis of 1H-tetrazoles and asymmetric biphenyls
Source: Sci Rep. 2025 Apr 15;15:12875. doi: 10.1038/s41598-025-95922-x (PMC12000388; doi:10.1038/s41598-025-95922-x)
Supplement: Supplementary file 1 — Supplementary Information. [file 41598_2025_95922_MOESM1_ESM.docx]

**Fe_3_O_4_@SiO_2_-LY-C-D-Pd as a new, effective, and magnetically recoverable catalyst for the synthesis of 1H-tetrazoles and asymmetric biphenyls**

Shelesh Krishna Saraswat ^1*^, Ahmed M. Naglah^2^, Jayanti Makasana^3^, Hamidah Abu Bakar^4^, Suhas Ballal^5^, Munthar Kadhim Abosaoda^6^, Kavitha V^7^, Lakshay Bareja^8^, Pushpa Negi Bhakuni^9,10^, Ojas Prakashbhai Doshi^11^

^1^Department of electronics and communication engineering, GLA University, Mathura-281406, INDIA. Email: ssheleshkrishna@gmail.com

^2^Department of Pharmaceutical Chemistry, College of Pharmacy, King Saud University, P.O. BOX 2457, 11451 Riyadh, Saudi Arabia

^3^Marwadi University Research Center, Department of Chemistry, Faculty of Science

Marwadi University, Rajkot-360003, Gujarat, India

^4^Management and Science University, Management and Science University

0000-0002-2775-0085

^5^Department of Chemistry and Biochemistry, School of Sciences, JAIN (Deemed to be University), Bangalore, Karnataka, India

^6^College of pharmacy, the Islamic University, Najaf, Iraq

^6^College of pharmacy, the Islamic University of Al Diwaniyah, Al Diwaniyah, Iraq

^7^Department of CHEMISTRY, Sathyabama Institute of Science and Technology, Chennai, Tamil Nadu, India

^8^Centre for Research Impact & Outcome, Chitkara University Institute of Engineering and Technology, Chitkara University, Rajpura, 140401, Punjab, India

^9^Department of Allied Science, Graphic Era Hill University, Bhimtal, Uttarakhand-248002, India.

^10^Graphic Era Deemed to be University, Dehradun, Uttarakhand, India.

^11^Arnold and Marie Schwartz College of Pharmacy and Health Sciences, Long Island University, Brooklyn, NY, USA

**Characterization data of compounds**

The 1 H NMR (DMSO-d_6_, 400 MHz) spectra were recorded using BRUKER AVANCE instruments in DMSO-d_6_ as solvent.

**1,1'-Biphenyl (Table 2, entry 1)**:

^1^H NMR (400 MHz, DMSO-d_6_): δ_H_= 7.0 (d, 5H), 6.83 (d, 5H) ppm. M.p. 68–69 °C.

**4-Methoxy-1,1'-biphenyl (Table 2, entry 6)**:

^1^H NMR (400 MHz, DMSO-d_6_): δ_H_= 7.2 (s, 2H), 7.18 (s, 2H), 7.10 (s, 3H), 6.93 (d, 2H), 4.09 (d, 3H) ppm. M.p. 80–82 °C.

**[1,1'-Biphenyl]-4-amine (Table 2, entry 5)**:

^1^H NMR (400 MHz, DMSO-d_6_): δ_H_= 7.81 (s, 1H), 7.67 (d, 2H), 7.43 (d, 2H), 7.17 (s, 4H), 4.91 (d, 2H) ppm. M.p. 54–56 °C.

**4-Nitro-1,1'-biphenyl (Table 2, entry 3)**:

^1^H NMR (400 MHz, DMSO-d_6_): δ_H_= 7.76 (d, 1H), 7.67 (d, 2H), 7.62 (d, 2H), 7.40 (d, 2H), 7.25 (s, 2H) ppm. M.p. 107–109 °C.

**5-(2-Chlorophenyl)-1H-tetrazole(Table 4, entry 4):**

 ^1^H NMR (400 MHz, DMSO-d_6_, ppm) δ = 7.24 (d, 1H, *Ar*), 7.12 (m, 3H, Ar), 4.80 (s, 1H, *NH*). M.p. 130–132 °C.

**2-(1H-Tetrazol-5-yl)phenol (Table 4, entry 6):**

 ^1^H NMR (400 MHz, DMSO-d_6_, ppm) δ = 10.27 (s, 1H, *OH*), 7.63 (m, 2H, *Ar*), 7.30 (m, 2H, Ar), 5.96 (s, 1H, NH). M.p. 224–226 °C.

**5-(4-Bromophenyl)-1H-tetrazole (Table 4, entry 9):**

^1^H NMR (400 MHz, DMSO-d_6_, ppm) δ = 7.63 (m, 2H, *Ar*), 7.39 (m, 2H, *Ar*), 5.95 (s, 1H, *NH*). M.p. 264–266 °C.

**3-Methoxy-1,1'-biphenyl (Table 2, entry 8)**:

^1^H NMR (400 MHz, DMSO-d_6_): δ_H_= 7.0 (m, 4H), 6.9 (m, 5H), 3.9 (s, 3H), ppm. M. p. oil

**4-Methyl-1,1'-biphenyl(Table 2, entry 2)**:

^1^H NMR (400 MHz, DMSO-d_6_): δ_H_= 7.4 (m, 4H), 7.1 (m, 5H), 1.1 (d, 3H), ppm. M.p. 43–45 °C.

**3-Nitro-1,1'-biphenyl (Table 2, entry 4)**:

^1^H NMR (400 MHz, DMSO-d_6_): δ_H_= 7.4 (m, 1H), 7.3 (m, 2H), 7.2 (m, 2H), 7.1 (m, 3H), ppm. M.p. 106–108 °C.

**1,1'-Biphenyl (Table 2, entry 9)**:

^1^H NMR (400 MHz, DMSO-d_6_): δ_H_= 7.4 (m, 10H), ppm. M.p. 68–69 °C.

**3-Methyl-1,1'-biphenyl (Table 2, entry 7)**:

^1^H NMR (400 MHz, DMSO-d_6_): δ_H_= 7.3 (m, 3H), 7.1 (m, 2H), 7.0 (m, 4H), 0.91 (s, 3H), ppm. M. p. oil

**4-Nitro-1,1'-biphenyl (Table 2, entry 12)**:

^1^H NMR (400 MHz, DMSO-d_6_): δ_H_= 7.2 (m, 9H), ppm. M.p. 107–109 °C.

**1,1'-Biphenyl (Table 2, entry 11)**:

^1^H NMR (400 MHz, DMSO-d_6_): δ_H_= 7.4 (m, 10H), ppm. M.p. 68–69 °C. M.p. 68–69 °C.

**4-Methoxy-1,1'-biphenyl (Table 2, entry 10)**:

^1^H NMR (400 MHz, DMSO-d_6_): δ_H_= 6.9 (m, 4H), 6.7 (m, 4H), 3.9 (m, 3H), ppm. M.p. 80–82 °C.

**3-Phenylpyridine (Table 2, entry 13)**:

^1^H NMR (400 MHz, DMSO-d_6_): δ_H_= 8.1 (m, 6H), 7.8 (m, 3H), ppm. M. p. Yellow oil

**5-(4-Chlorophenyl)-1H-tetrazole (Table 4, entry 1):**

 ^1^H NMR (400 MHz, DMSO-d_6_, ppm) δ = 7.4 (m, 3H, *Ar*), 7.1 (m, 1H, *Ar*), 5.1 (s, 1H, *NH*). M.p. 260–262 °C.

**4-(1H-Tetrazol-5-yl)benzonitrile (Table 4, entry 7):**

^1^H NMR (400 MHz, DMSO-d_6_, ppm) δ = 7.4-7.6 (m, 4H, *Ar*), 5.8 (s, 1H, *NH*). M.p. 253–256 °C.

**5-(3-Nitrophenyl)-1H-tetrazole (Table 4, entry 3):**

 ^1^H NMR (400 MHz, DMSO-d_6_, ppm) δ = 7.3-7.7 (m, 4H, *Ar*), 5.1 (s, 1H, *NH*). M.p. 148–151 °C.

**5-(4-Nitrophenyl)-1H-tetrazole (Table 4, entry 5):**

^1^H NMR (400 MHz, DMSO-d_6_, ppm) δ = 7.7 (m, 2H, *Ar*), 7.5 (m, 1H, *Ar*), 7.3 (m, 1H, *Ar*), 5.6 (s, 1H, *NH*). M.p. 218–220 °C.

**4-(1H-tetrazol-5-yl)phenol (Table 4, entry 8):**

^1^H NMR (400 MHz, DMSO-d_6_, ppm) δ = 8.7 (s, 1H), 7.7 (m, 3H, *Ar*), 7.5 (m, 2H, *Ar*), 5.7 (s, 1H, *NH*). M.p. 231–233 °C.

**5-Phenyl-1H-tetrazole (Table 4, entry 2):**

 ^1^H NMR (400 MHz, DMSO-d_6_, ppm) δ = 7.3 (m, 3H, *Ar*), 7.0 (m, 2H, *Ar*), 5.6 (s, 1H, *NH*). M.p. 213–214 °C.


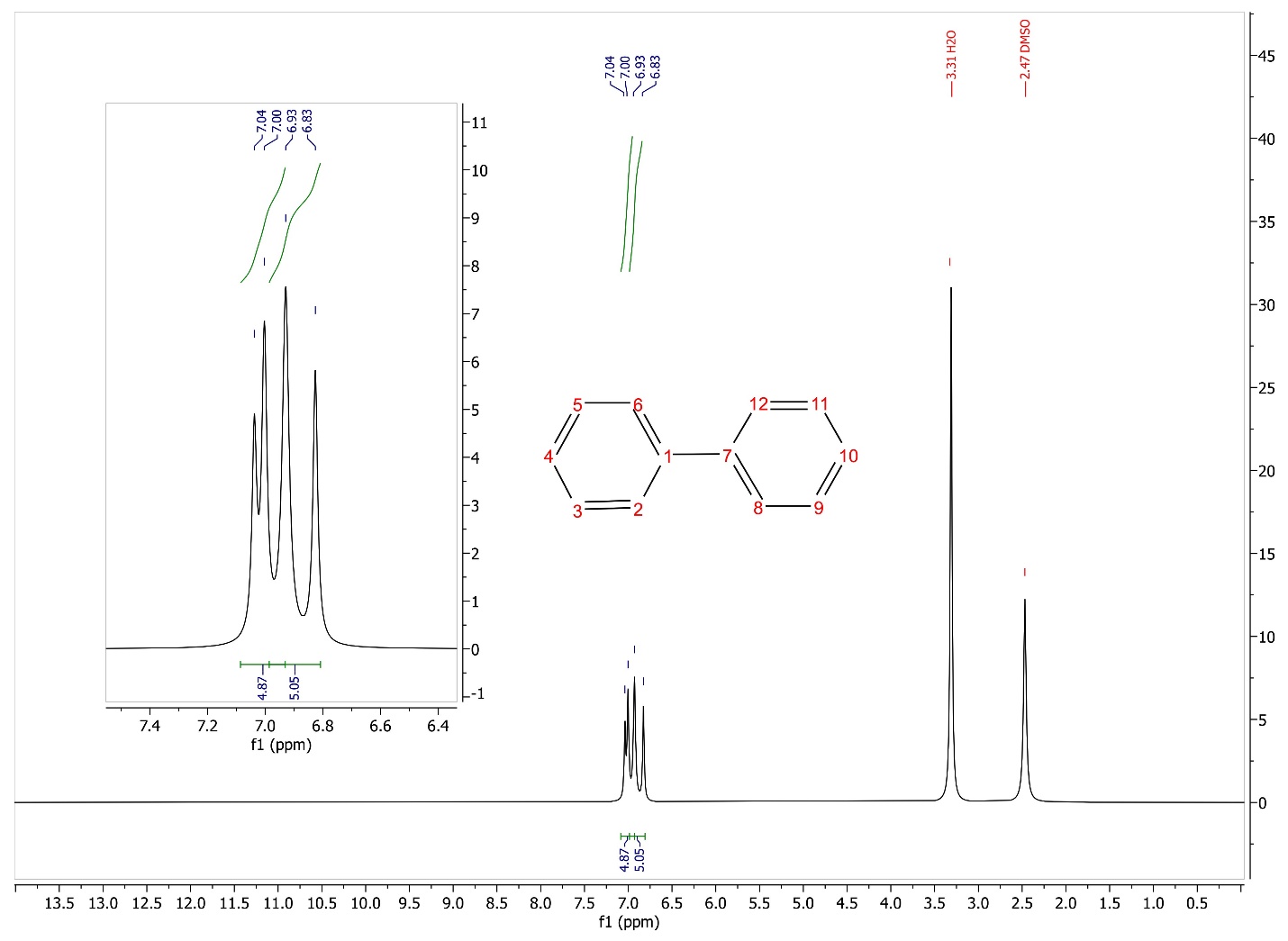


Figure S1. 1,1'-Biphenyl (Table 2, entry 1)


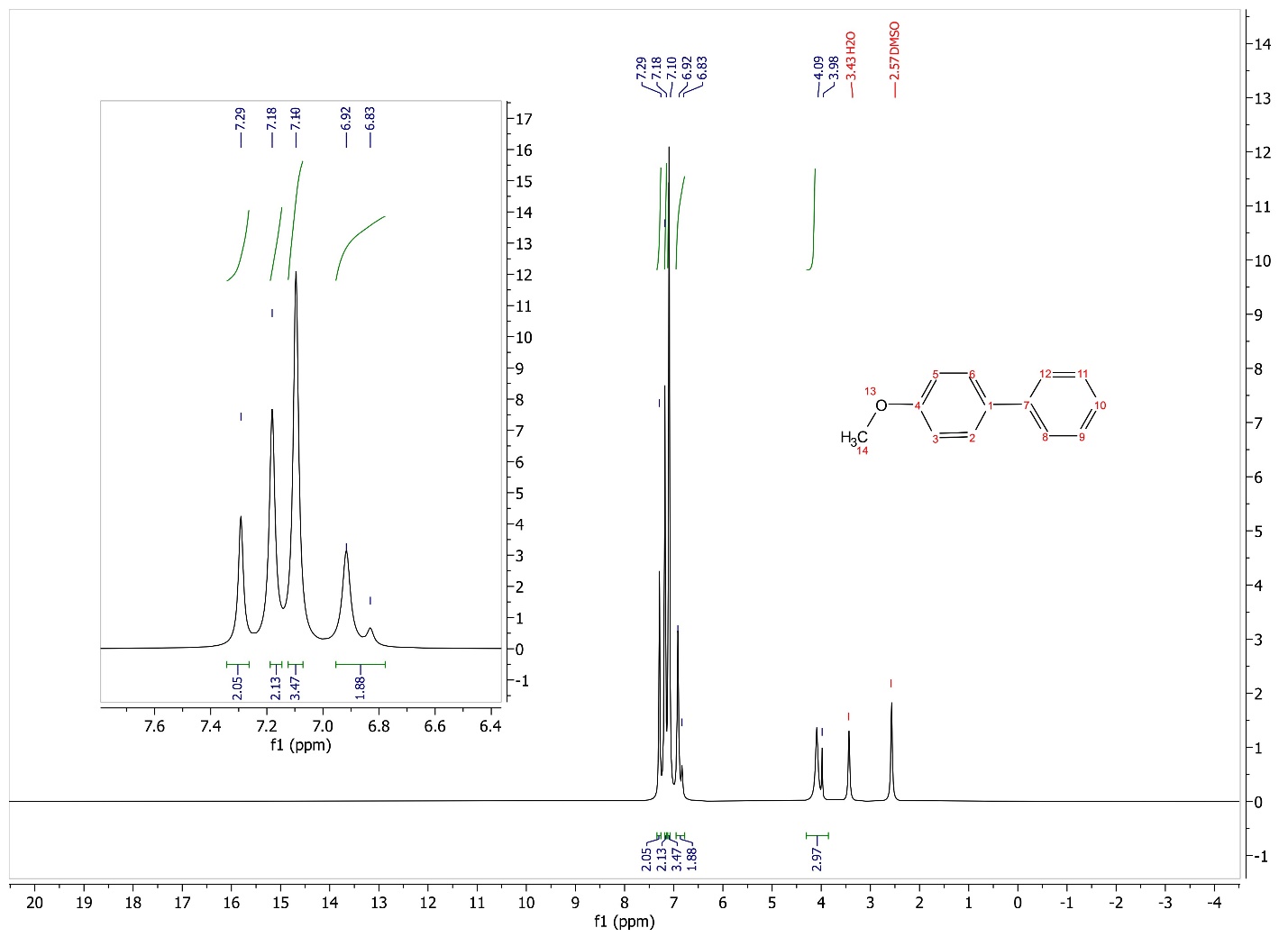


Figure S2. 4-Methoxy-1,1'-biphenyl (Table 2, entry 6)


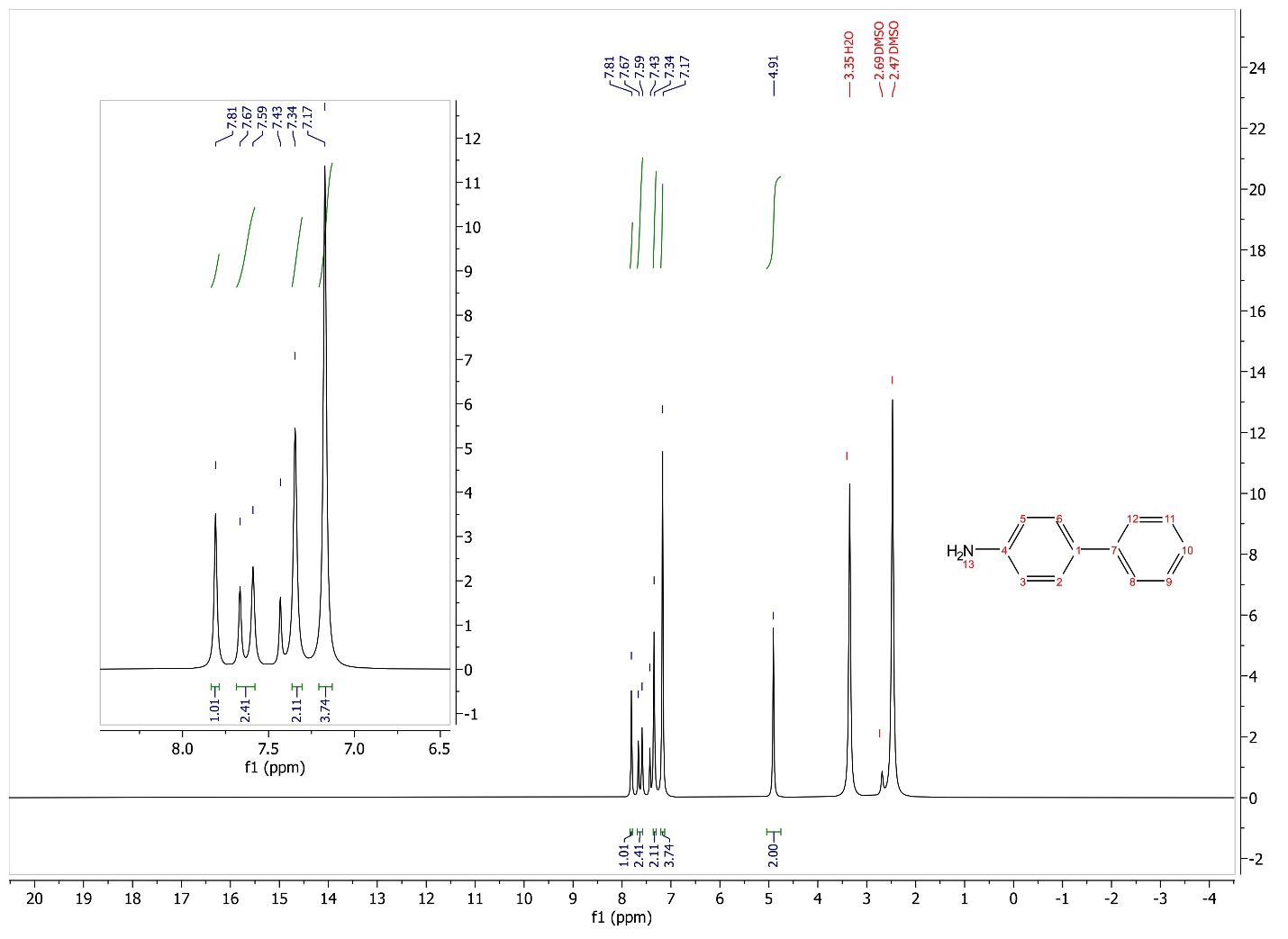


Figure S3. [1,1'-Biphenyl]-4-amine (Table 2, entry 5)


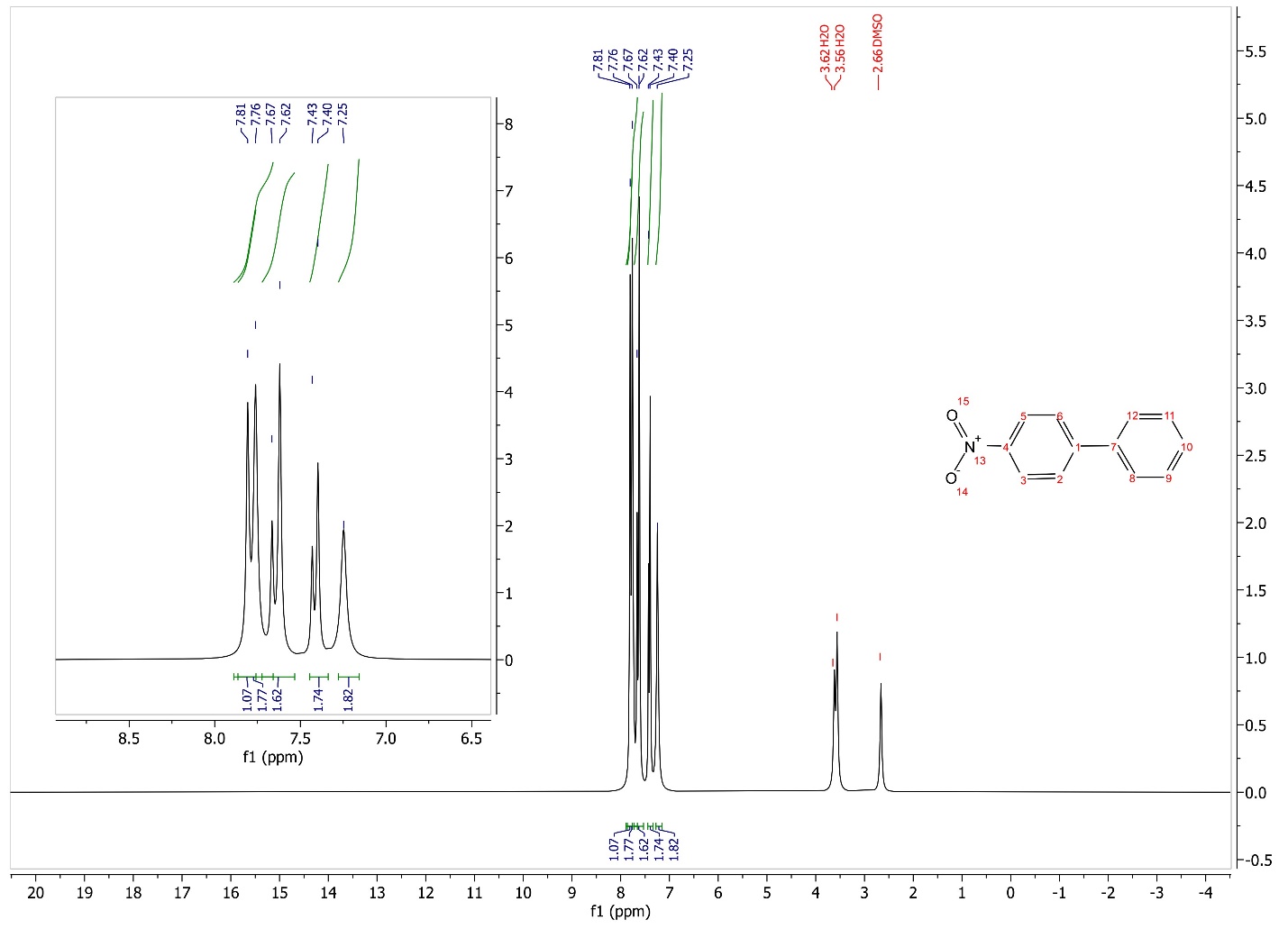


Figure S4. 4-Nitro-1,1'-biphenyl (Table 2, entry 3)


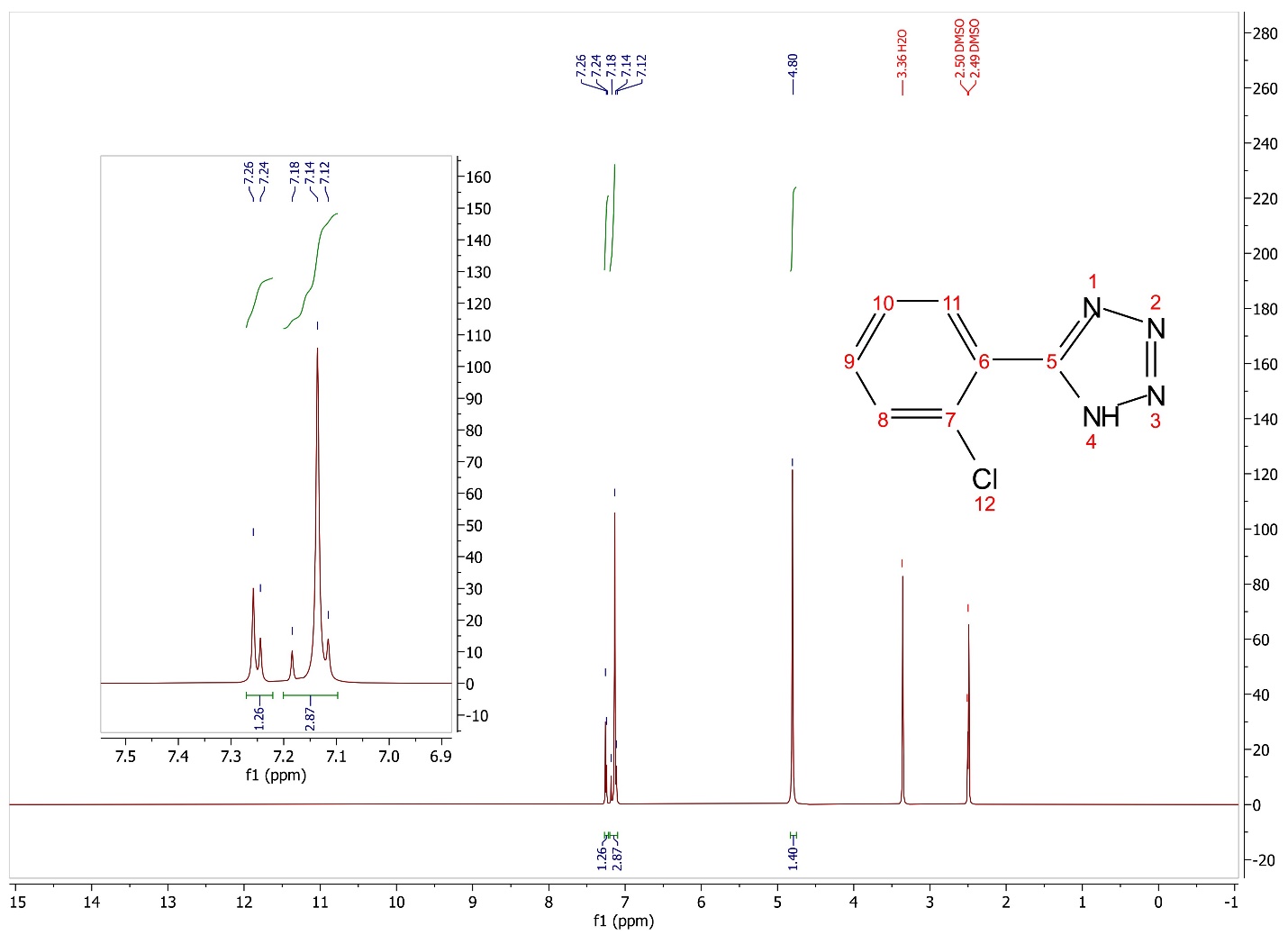


Figure S5. 5-(2-Chlorophenyl)-1H-tetrazole(Table 4, entry 4)


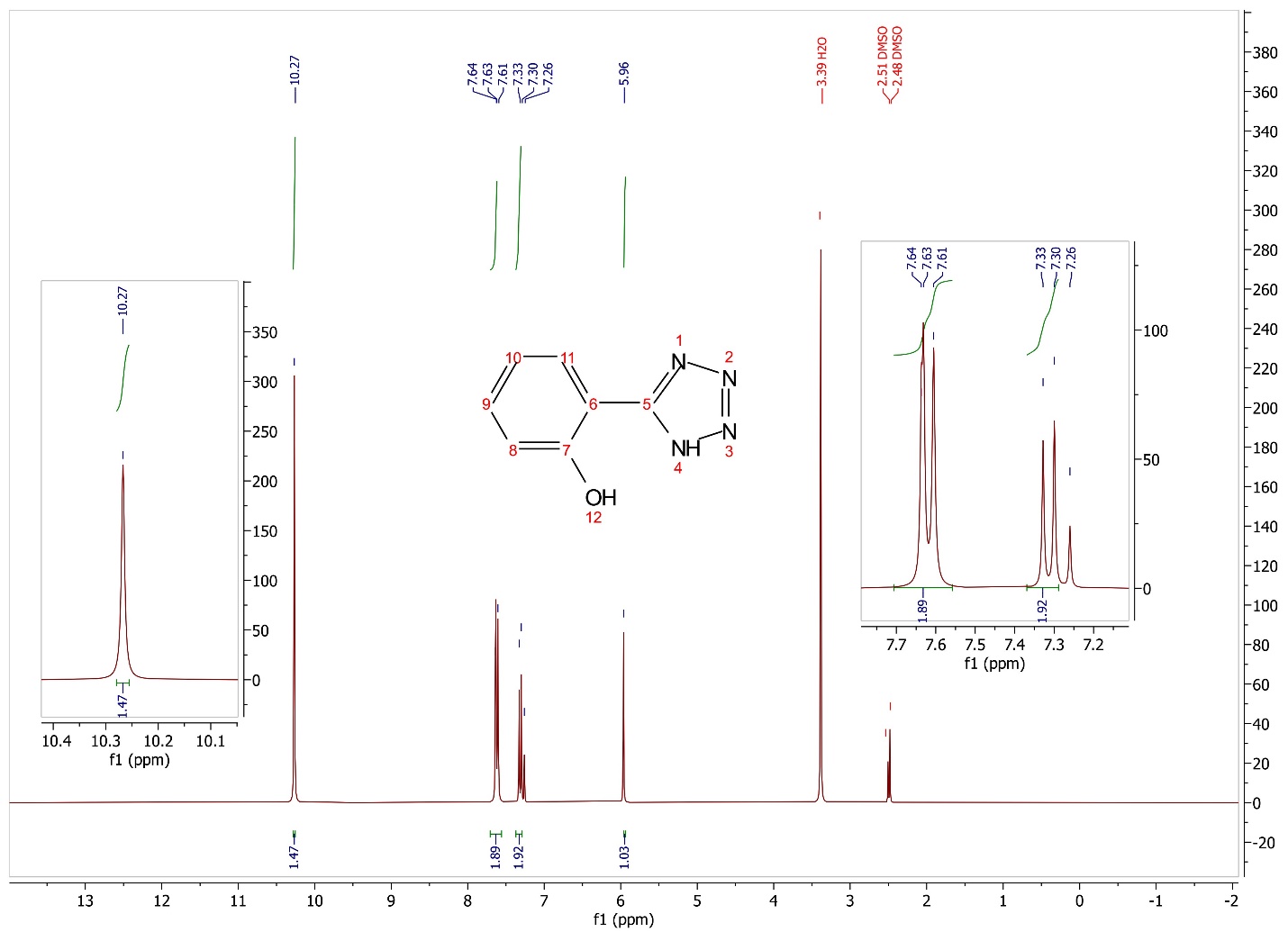


Figure S6. 2-(1H-Tetrazol-5-yl)phenol(Table 4, entry 6)


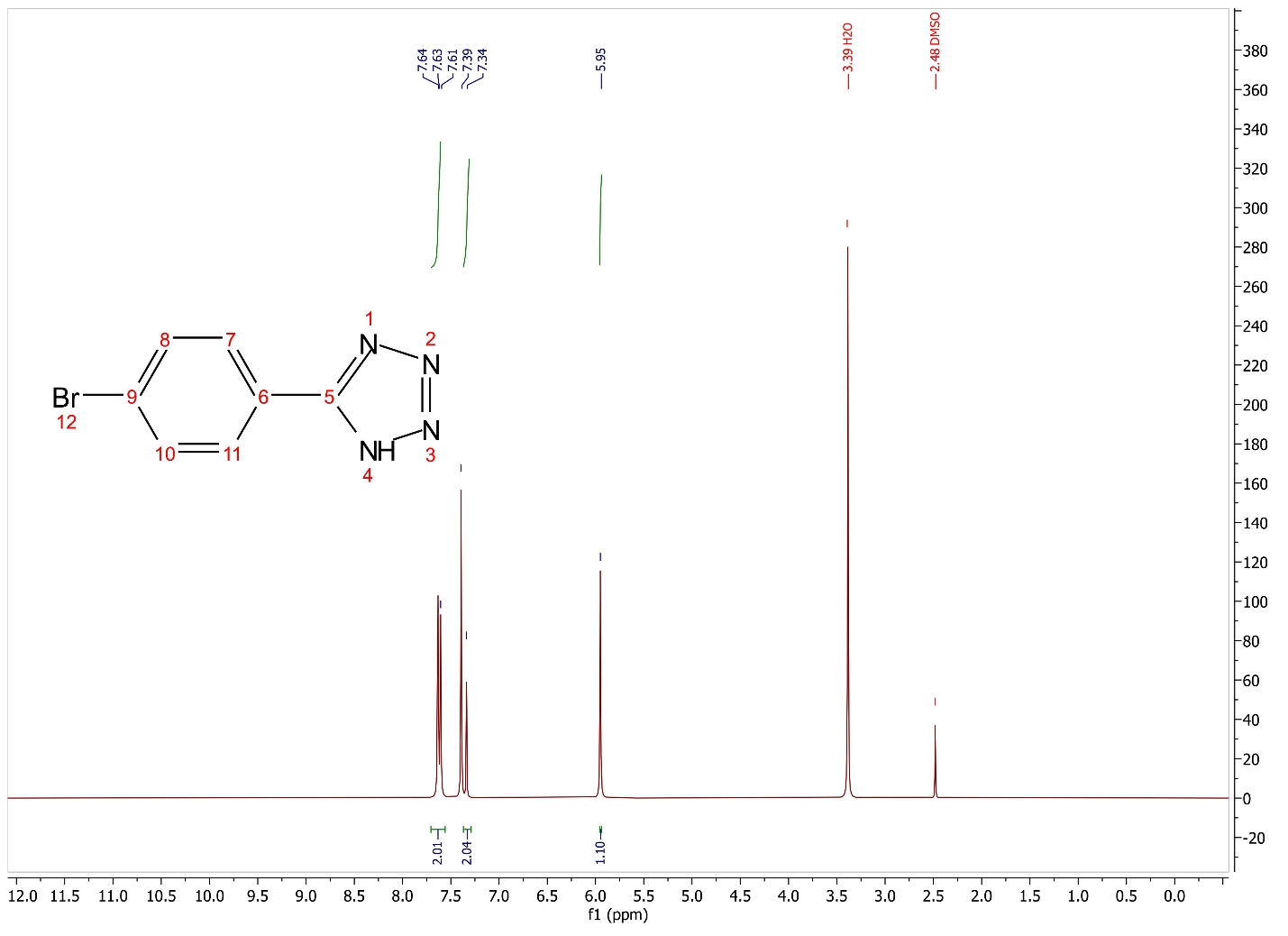


Figure S7. 5-(4-Bromophenyl)-1H-tetrazole (Table 4, entry 9)


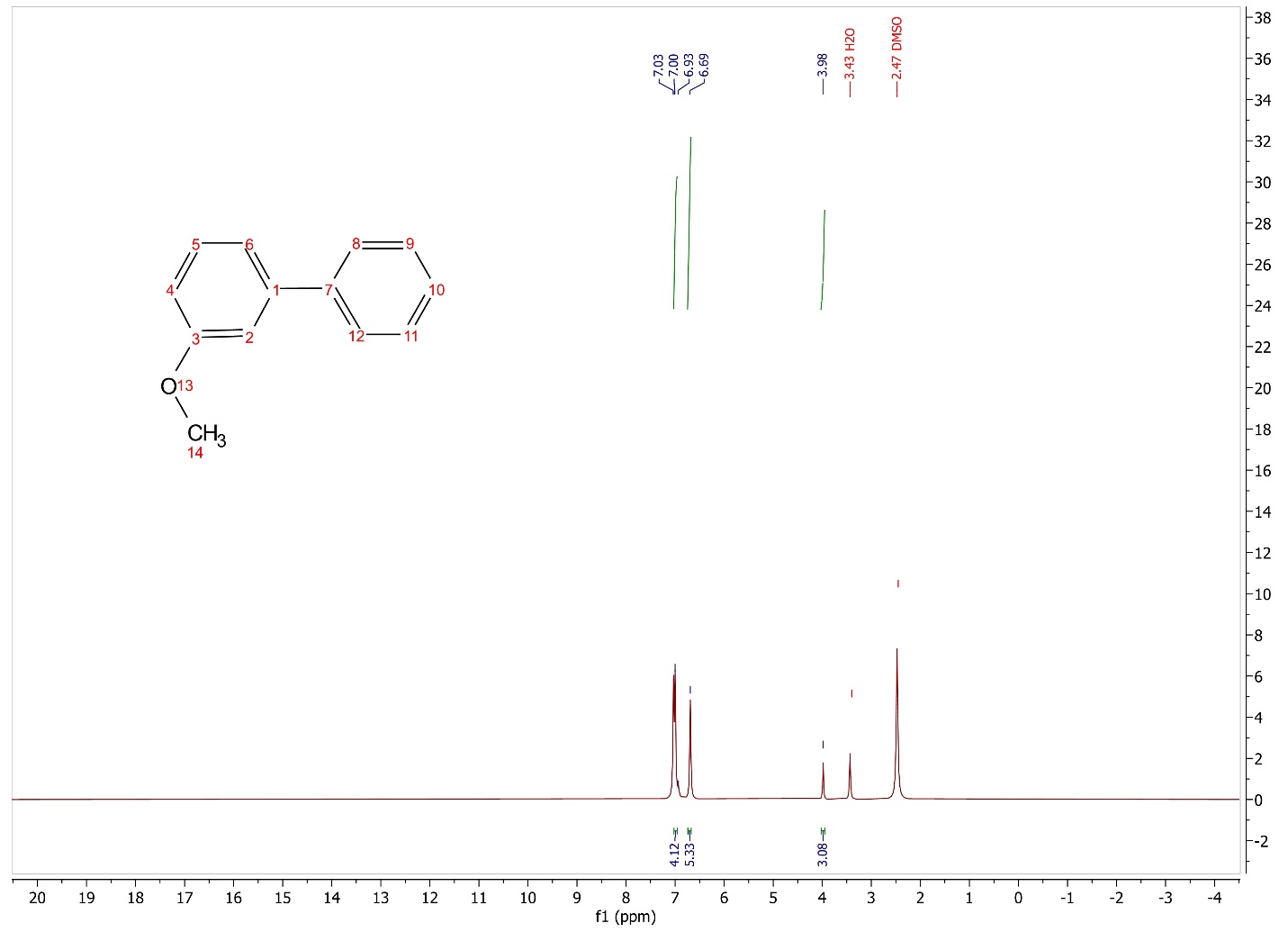


Figure S8. 3-Methoxy-1,1'-biphenyl (Table 2, entry 8)


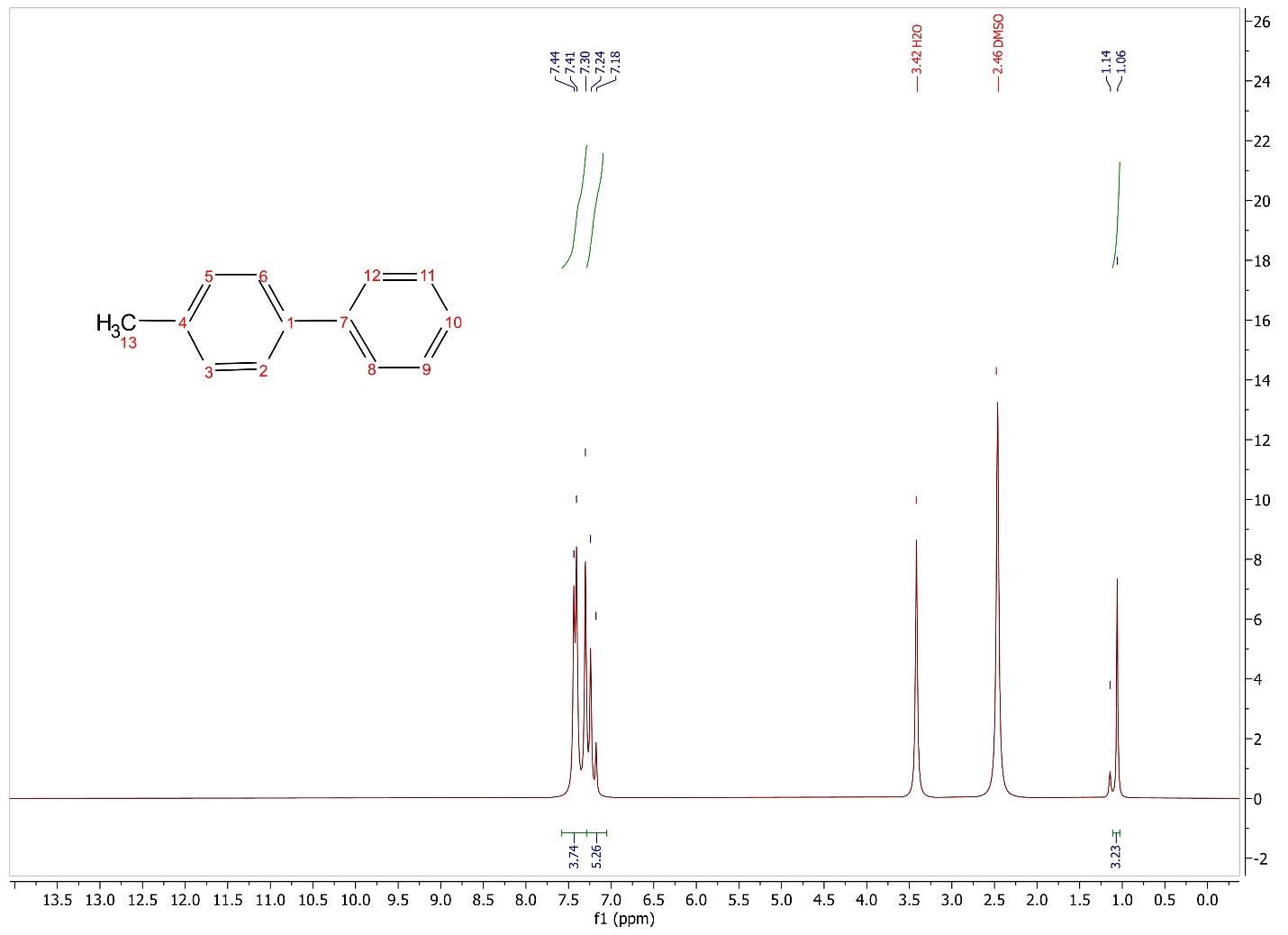


Figure S9. 4-Methyl-1,1'-biphenyl (Table 2, entry 2)


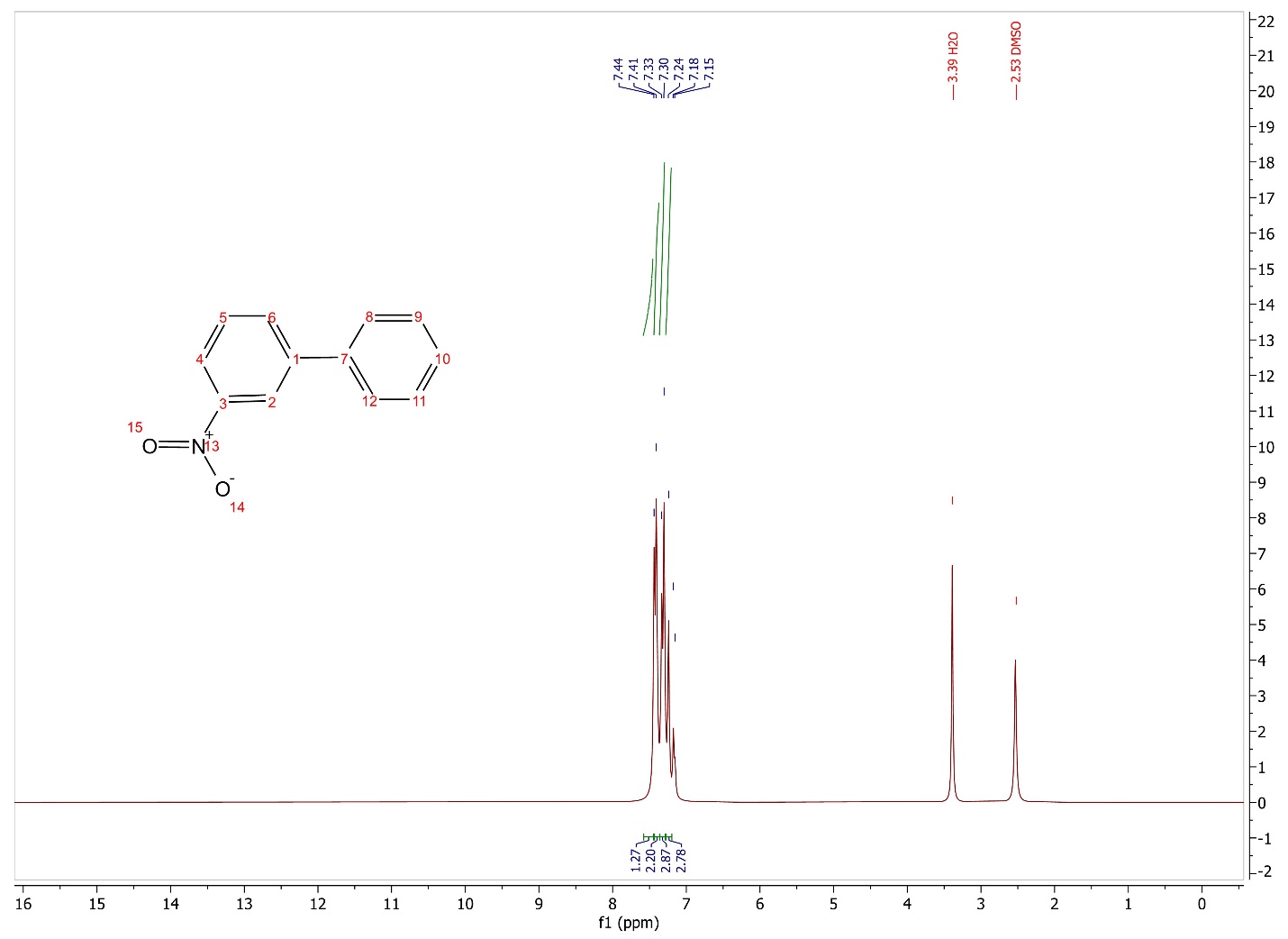


Figure S10. 3-Nitro-1,1'-biphenyl (Table 2, entry 4)


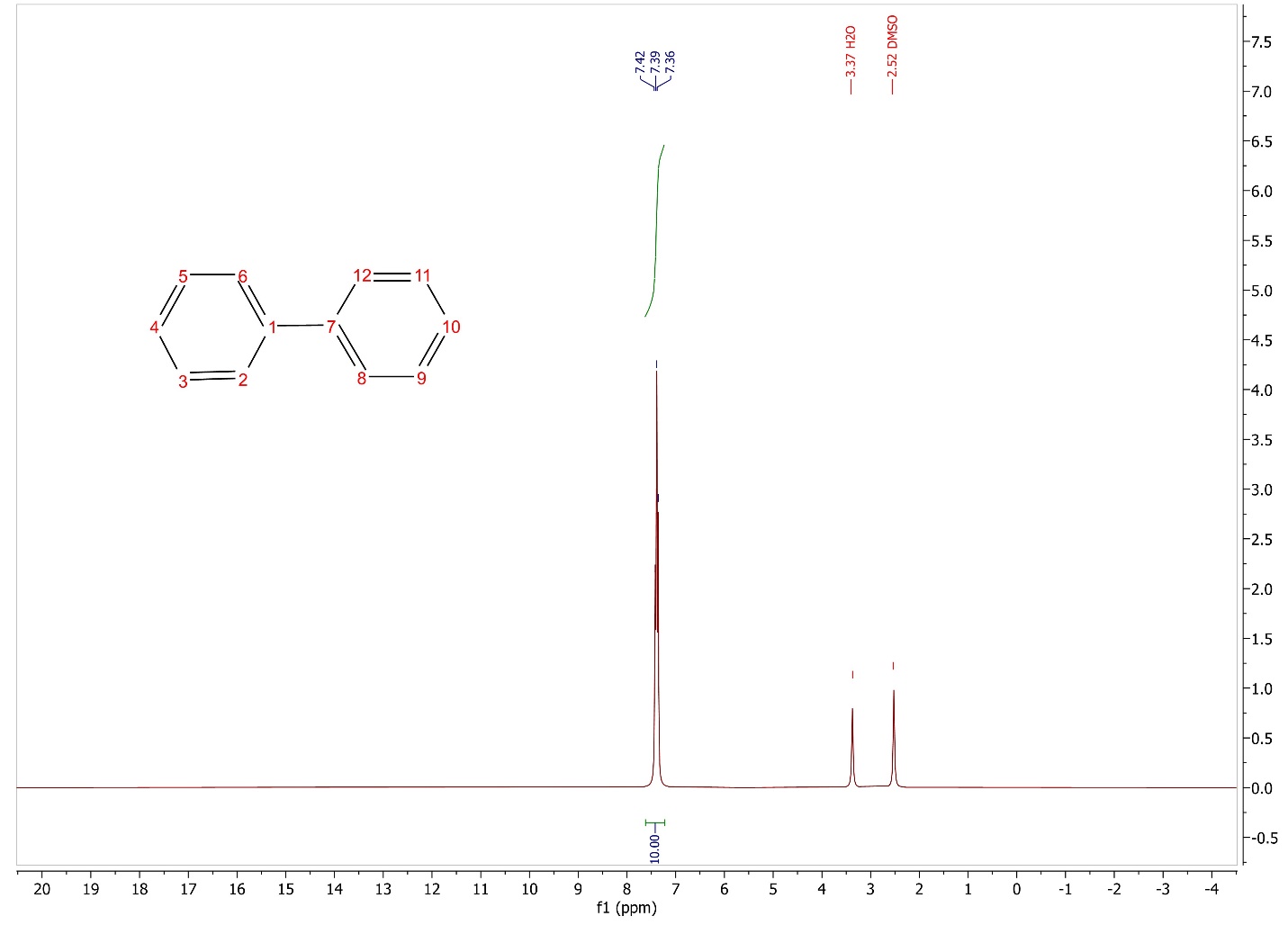


Figure S11. 1,1'-Biphenyl (Table 2, entry 9)


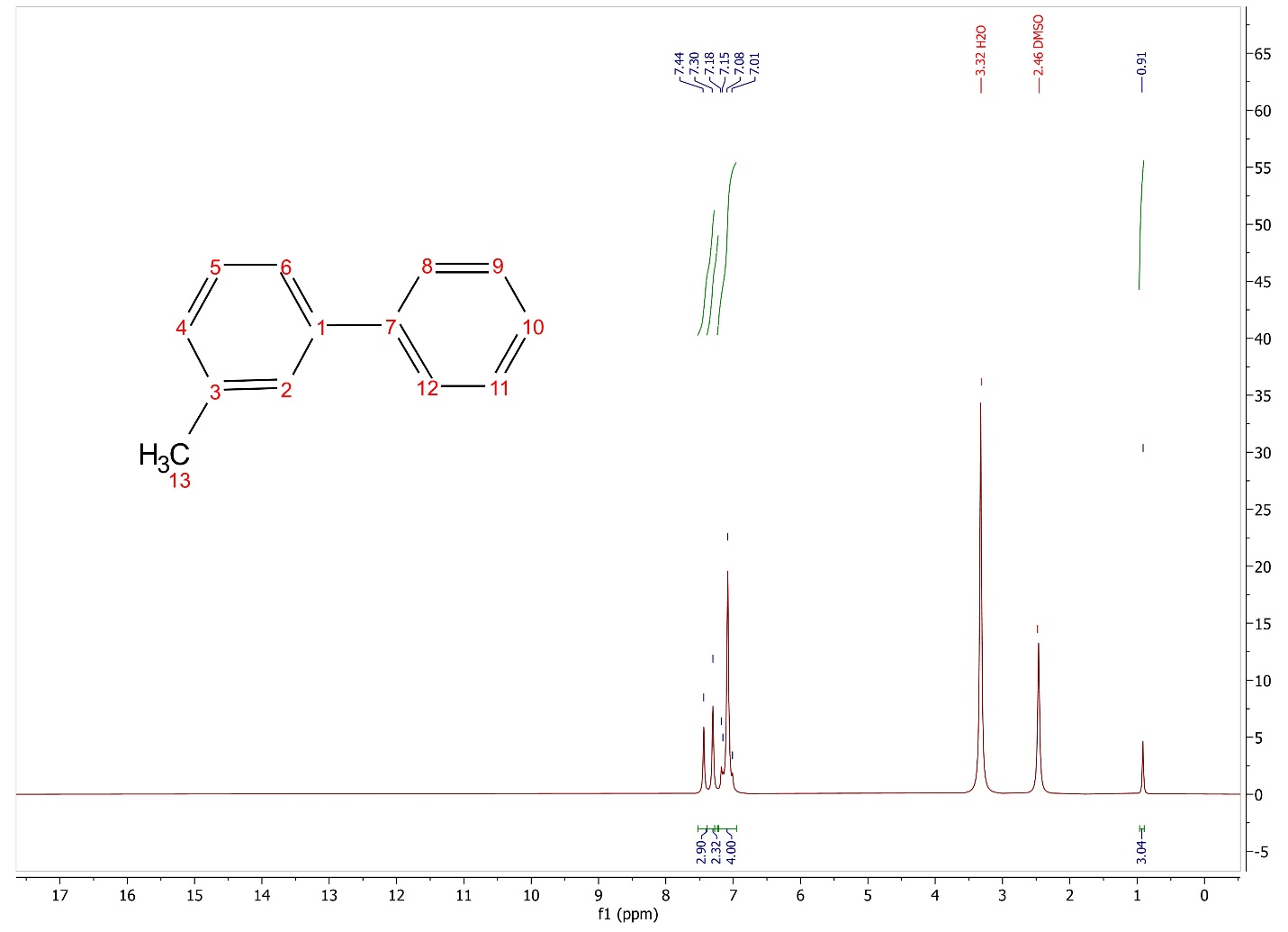


Figure S12. 3-Methyl-1,1'-biphenyl (Table 2, entry 7)


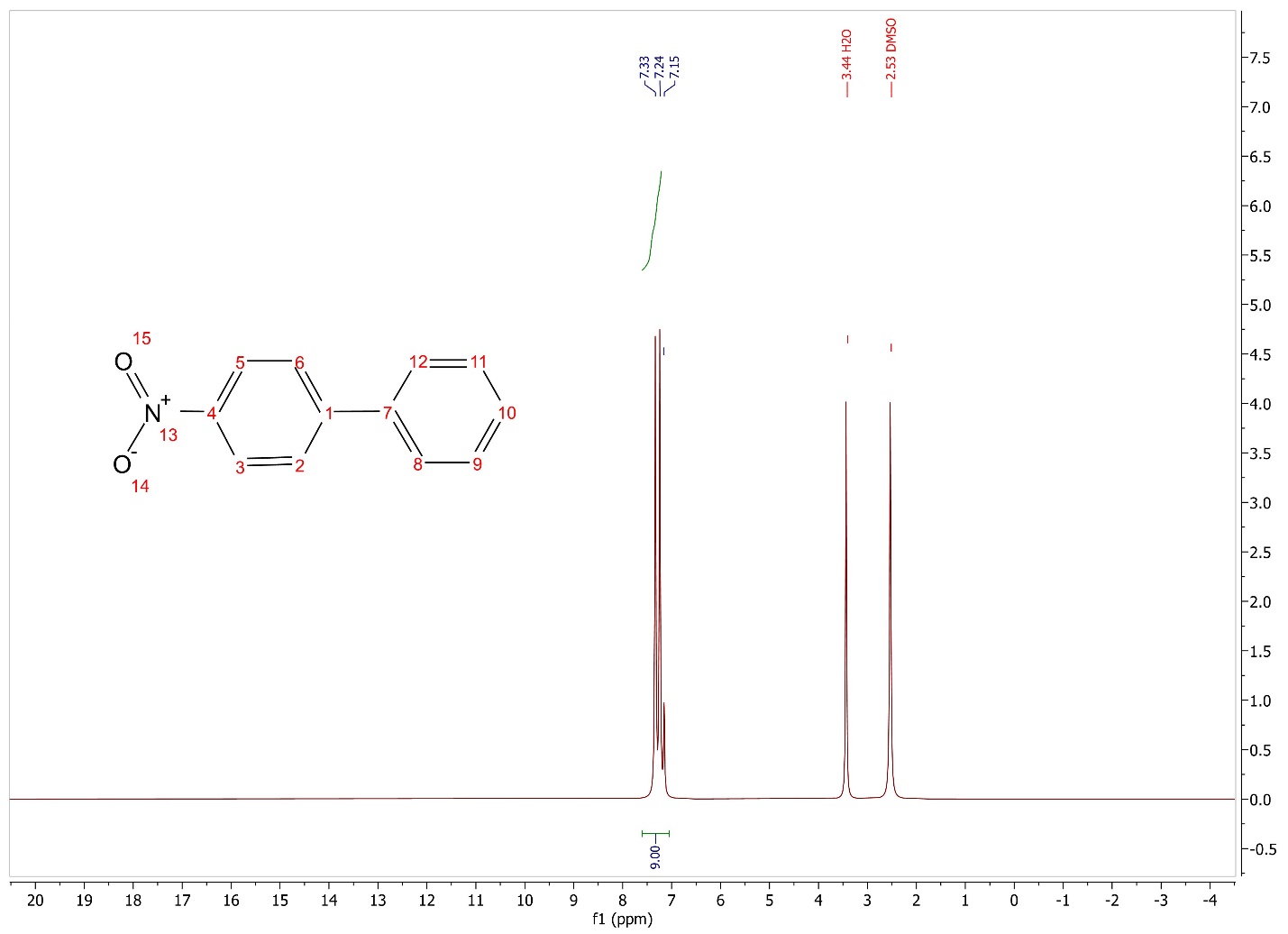


Figure S13. 4-Nitro-1,1'-biphenyl (Table 2, entry 12)


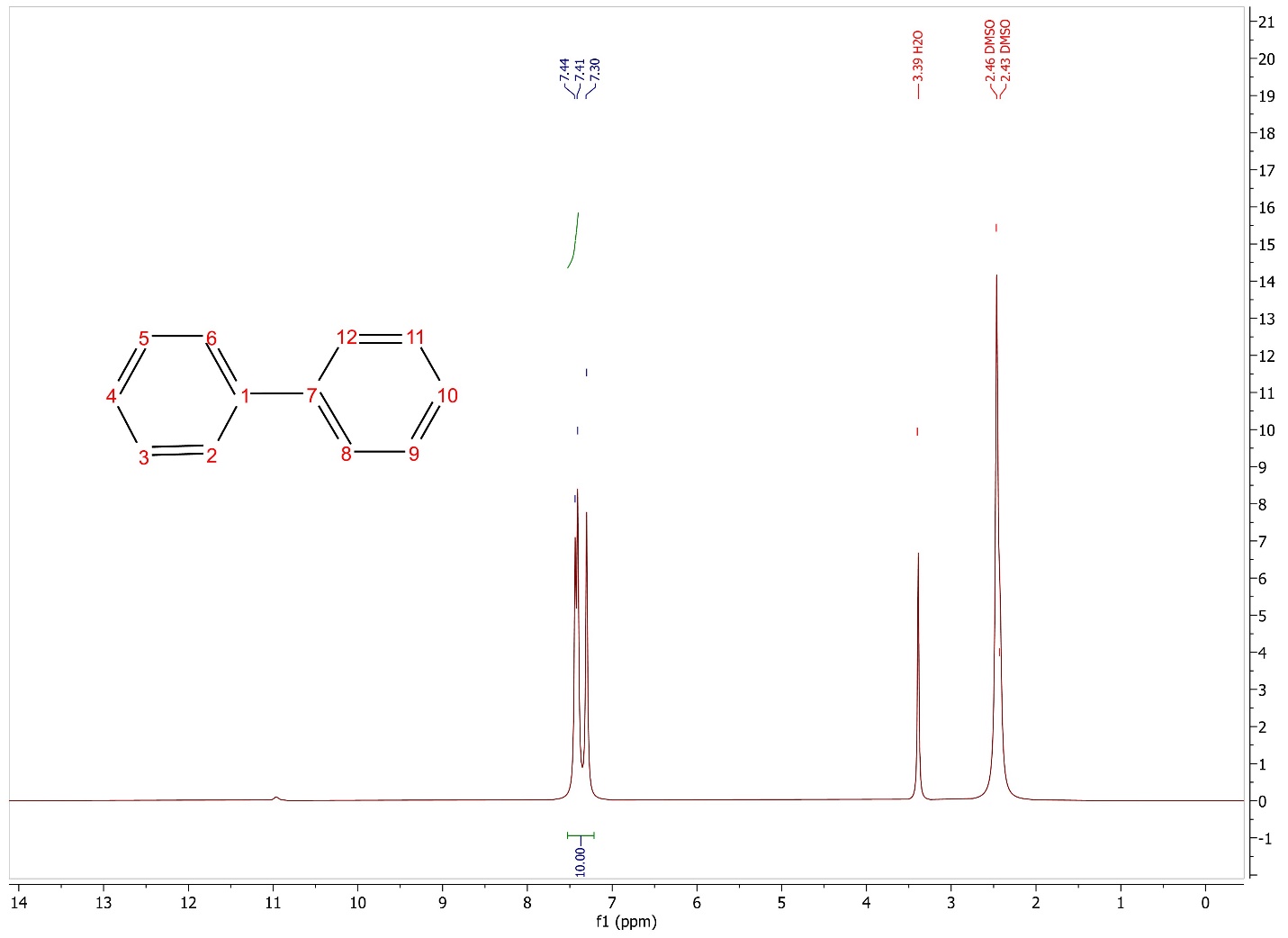


Figure S14. 1,1'-Biphenyl (Table 2, entry 11)


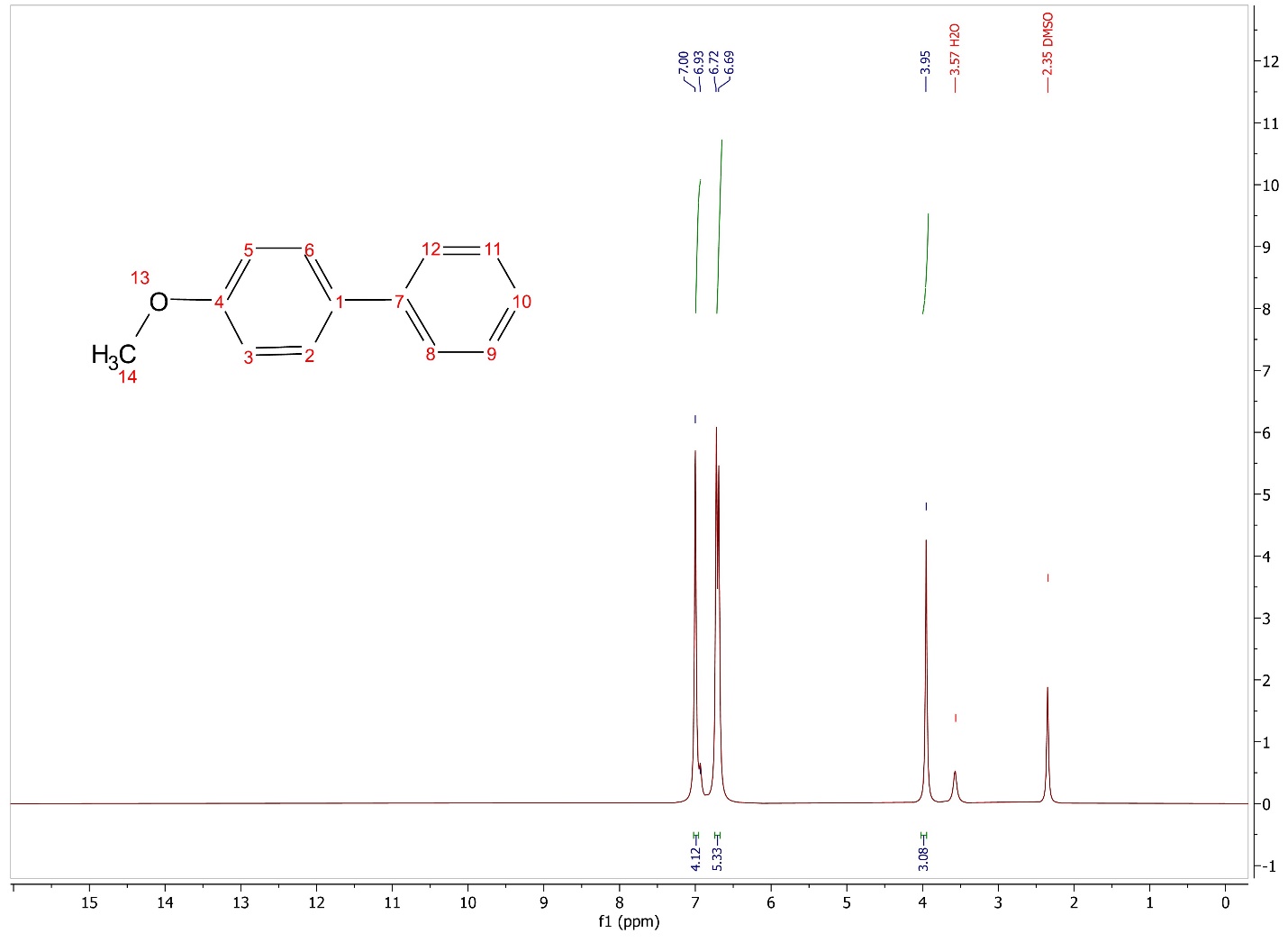


Figure S15. 4-Methoxy-1,1'-biphenyl (Table 2, entry 10)


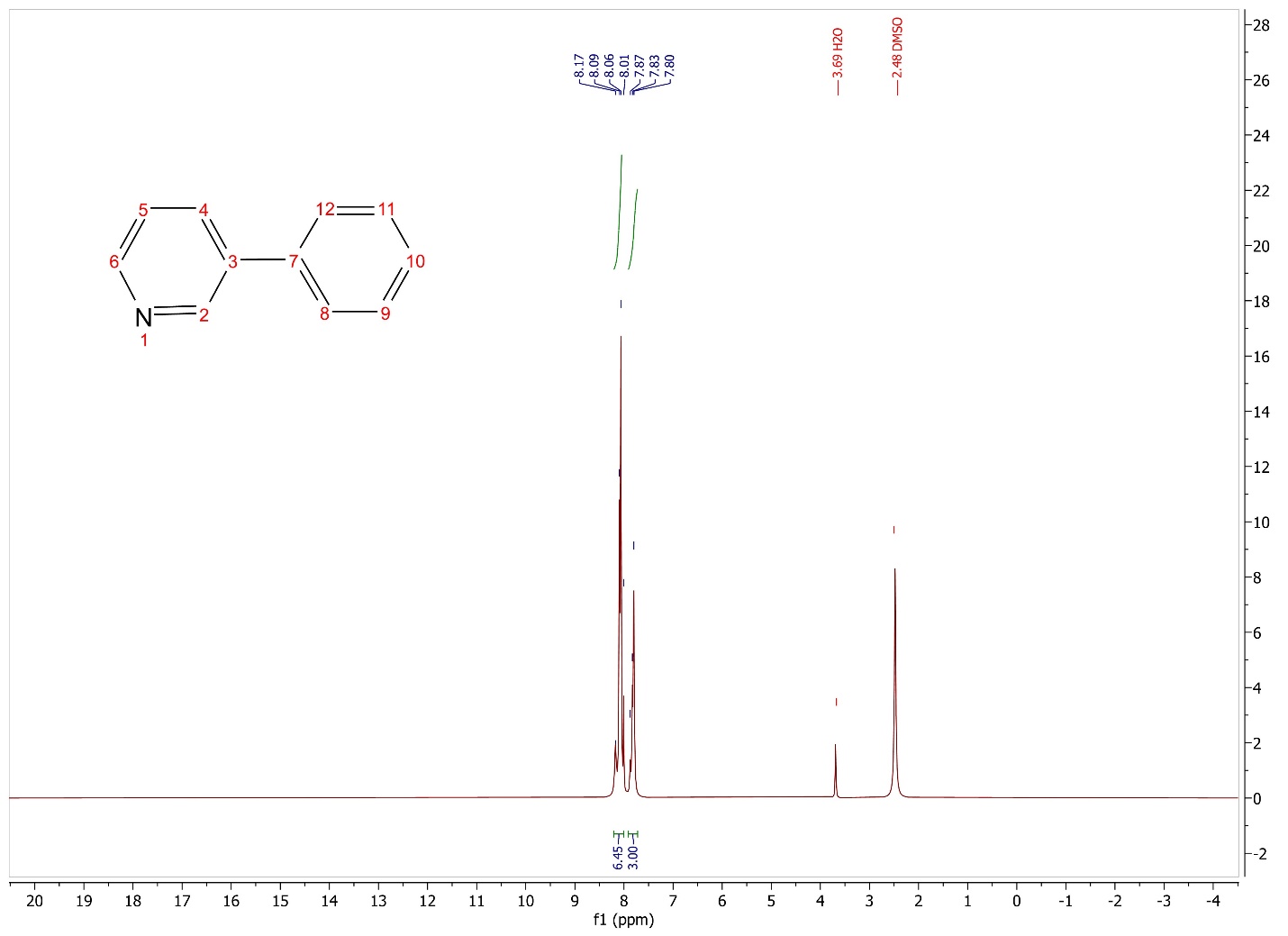


Figure S16. 3-Phenylpyridine (Table 2, entry 13)


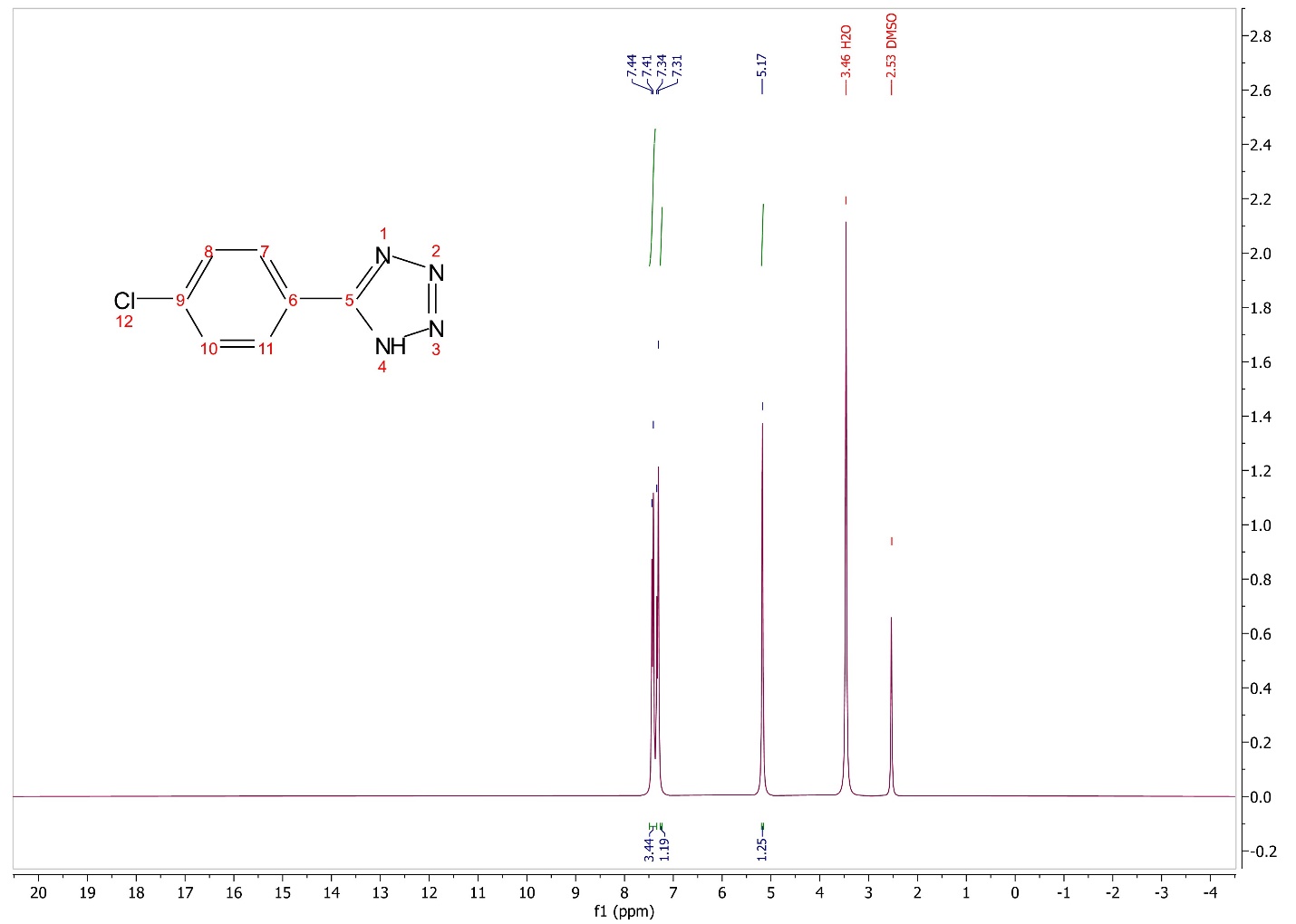


Figure S17. 5-(4-Chlorophenyl)-1H-tetrazole (Table 4, entry 1)


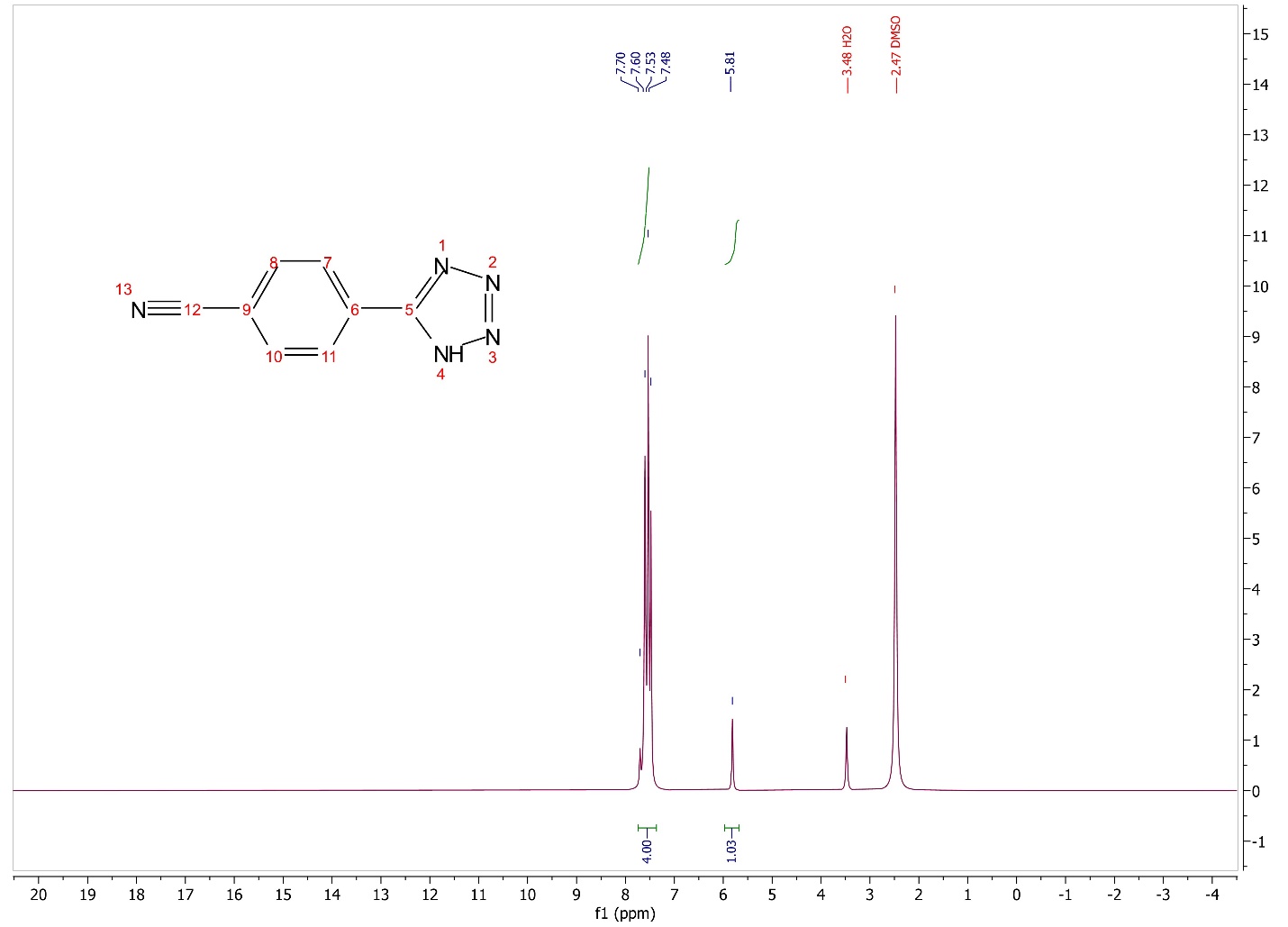


Figure S18. 4-(1H-Tetrazol-5-yl)benzonitrile (Table 4, entry 7)


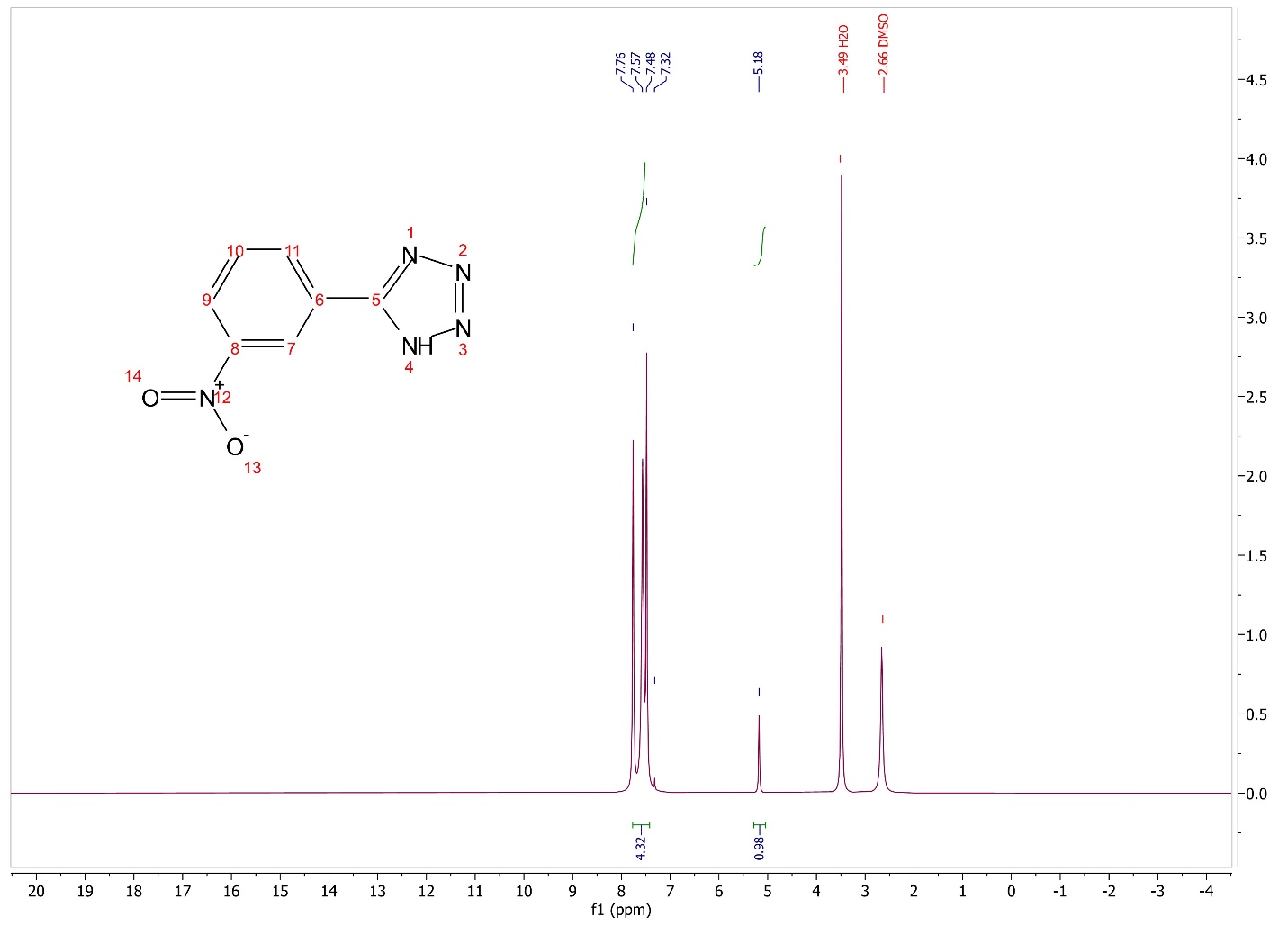


Figure S19. 5-(3-Nitrophenyl)-1H-tetrazole (Table 4, entry 3)


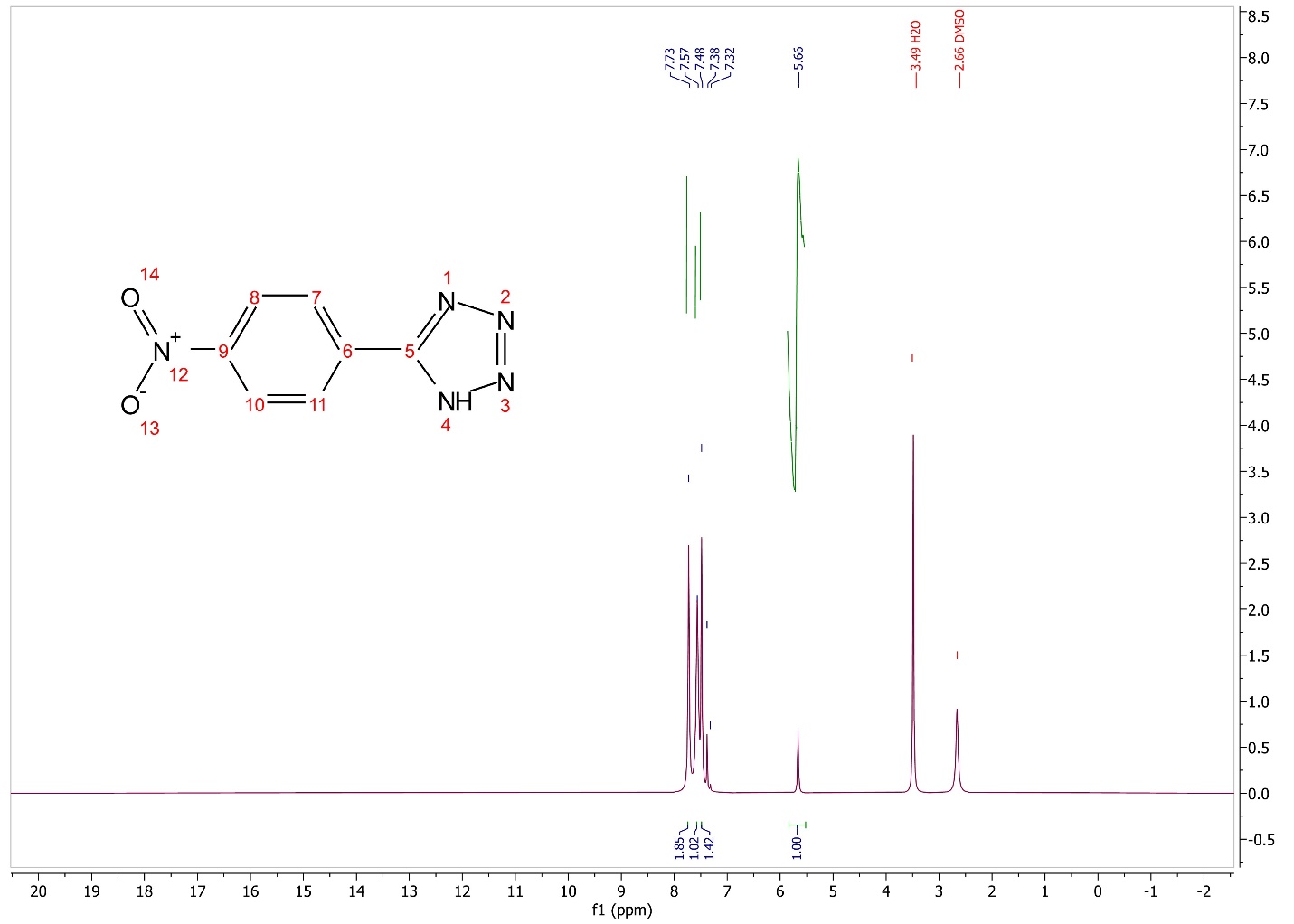


Figure S20. 5-(4-Nitrophenyl)-1H-tetrazole (Table 4, entry 5)


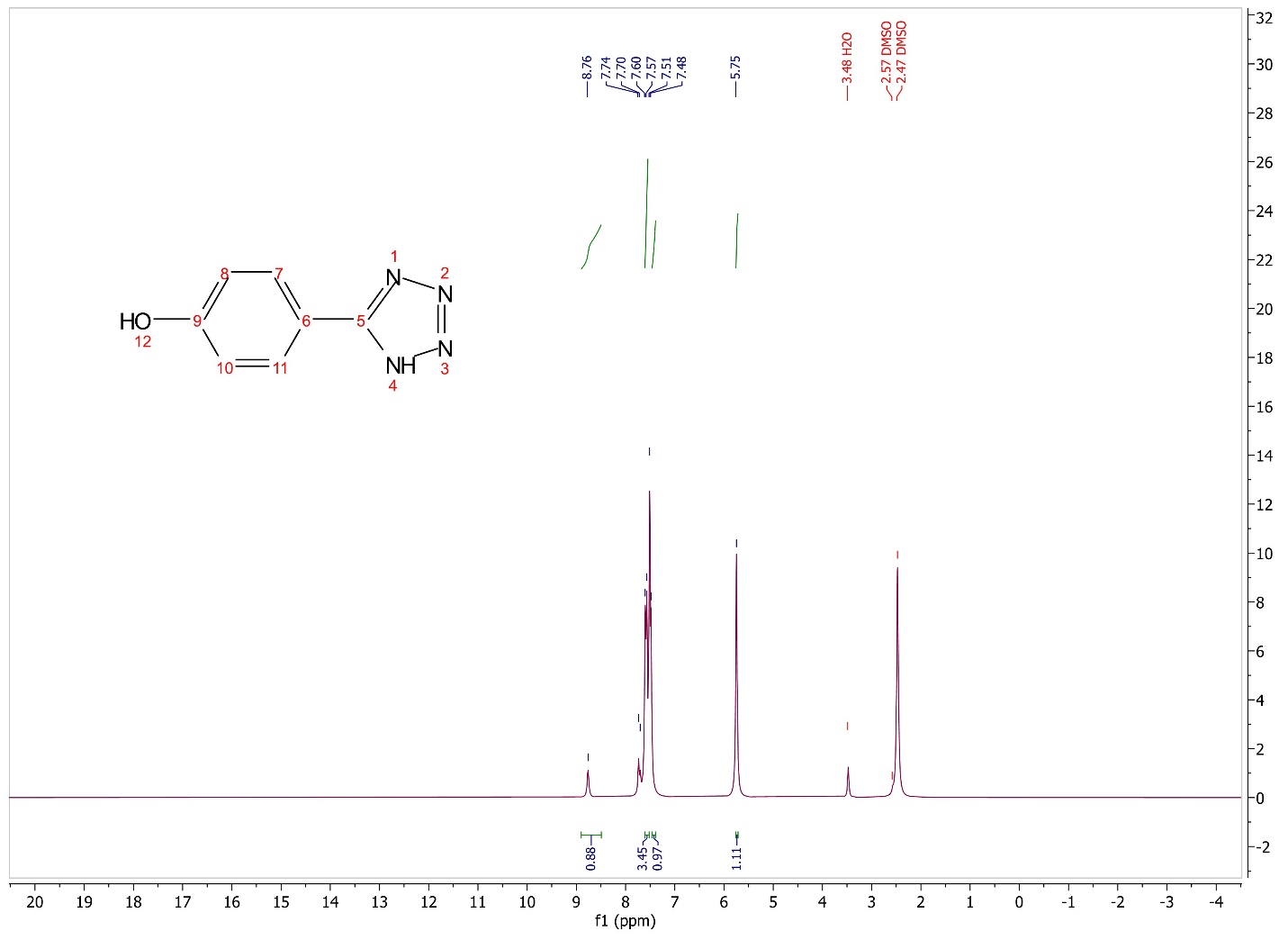


Figure S21. 4-(1H-Tetrazol-5-yl)phenol (Table 4, entry 8)


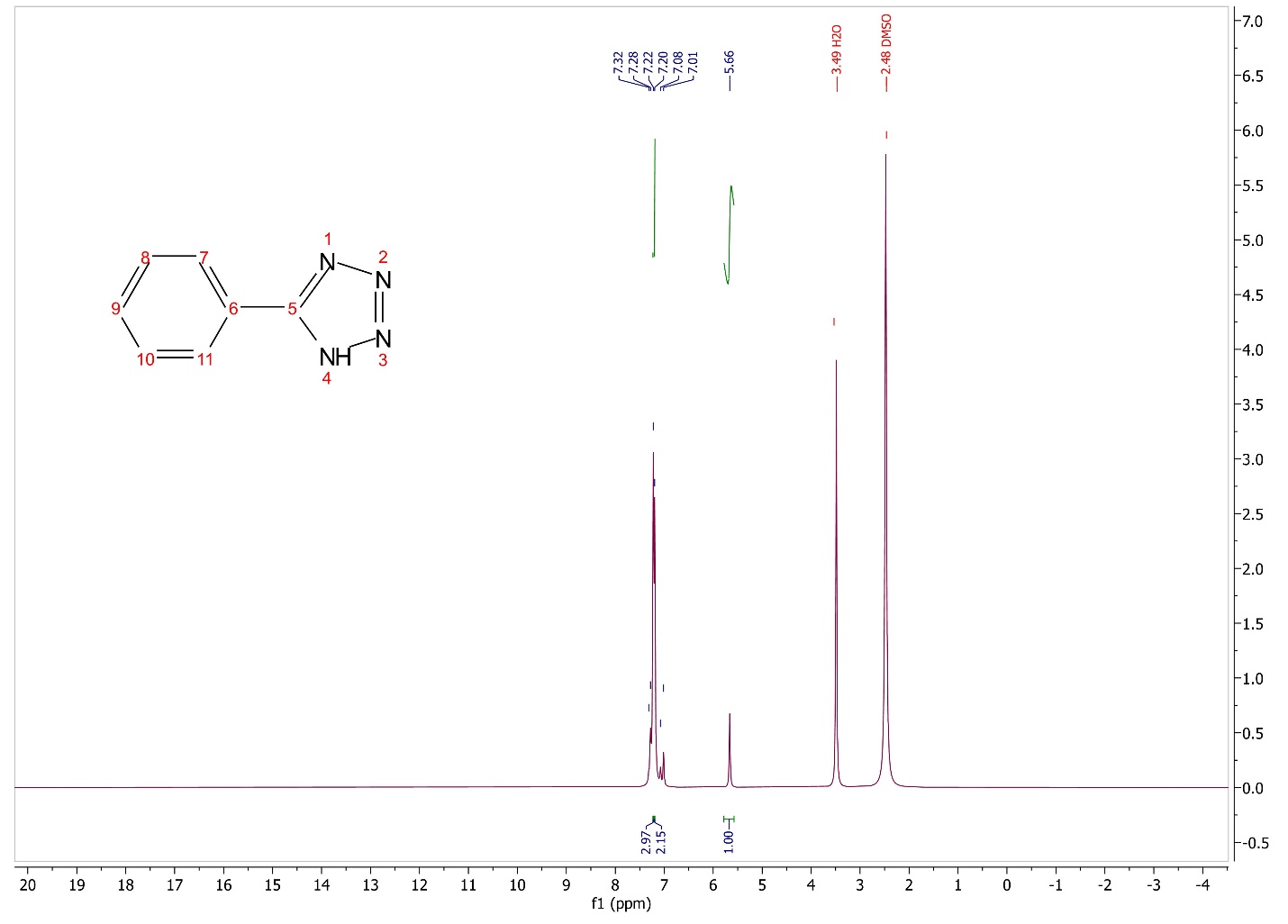


Figure S22. 5-Phenyl-1H-tetrazole (Table 4, entry 2)
